# Mutations in tumor signaling, metastases, and synthetic lethality establish distinct patterns

**DOI:** 10.1371/journal.pcbi.1013351

**Published:** 2025-08-04

**Authors:** Bengi Ruken Yavuz, Ugur Sahin, Hyunbum Jang, Ruth Nussinov, Nurcan Tuncbag

**Affiliations:** 1 Cancer Innovation Laboratory, National Cancer Institute, Frederick, Maryland, United States of America; 2 Department of Molecular Biology and Genetics, Koç University, Istanbul, Turkey; 3 Computational Structural Biology Section, Frederick National Laboratory for Cancer Research, Frederick, Maryland, United States of America; 4 Department of Human Molecular Genetics and Biochemistry, Sackler School of Medicine, Tel Aviv University, Tel Aviv, Israel; 5 Department of Chemical and Biological Engineering, College of Engineering, Koç University, Istanbul, Turkey; 6 School of Medicine, Koç University, Istanbul, Turkey; 7 Koc University Research Center for Translational Medicine (KUTTAM), Istanbul, Turkey; University of Connecticut School of Medicine, UNITED STATES OF AMERICA

## Abstract

Effective identification of oncogenic mutations is essential for diagnosis, forecasting resistance, and metastasis in remission. It is required for an optimal drug regimen. We develop a framework to discover mutations that *co-exist* in different oncoproteins, and those that are *excluded*, likely encoding oncogene-induced senescence. First, mapping the proteins onto pathways assists combinatorial drug selections and helps to detect metastases. Second, it provides the molecular basis for synthetic lethality, to date investigated at the genome level. Our pan-cancer profiles of ~60,000 tumor sequences, detect 3424 co-existing tumor-specific mutations. Mapping them onto pathways indicates that they preferentially promote specific primary tumors. We uncover metastatic mutations and provide metastatic breast-cancer markers. This work not only clarifies the mechanistic basis of intratumor mutational diversity but usefully reveals markers for metastasis in patients’ genomes and introduces a novel computational framework for detecting metastasis based on tumor mutational profiles. Mapping the mutations onto pathways provides an invaluable metastasis-targeting resource, guiding drug combinations.

## Author summary

Cancer is driven by combinations of mutations in specific proteins in certain cellular pathways; identifying these combinations can reveal new ways to treat the disease more effectively. We analyzed nearly 60,000 tumor genomes from a wide range of cancers to identify mutations that frequently occur together in different cancer driving proteins, as well as combinations that rarely appear in the same tumor. Co-occurring mutations may indicate signaling pathways that are prone to cancer growth and metastasis. In contrast, mutually exclusive mutations may point to processes like senescence or vulnerability to specific treatments. By mapping these mutation patterns onto known cellular pathways, we found thousands of tumor-specific mutation pairs, many linked to distinct cancer types. We also identified key mutations associated with metastasis, including markers specific to metastatic breast cancer. Our study introduces a new computational framework for detecting metastasis and guides drug selection based on the tumor mutational profile. This approach reframes tumor heterogeneity from cell signaling perspective and provides a resource for developing personalized cancer therapies.

## Introduction

Here we discover and interpret patterns of mutations in tumor signaling and metastases. Decades ago, it was proposed that cancer evolution requires more than a single mutation [1–4]. The minimal number has been debated since, and their cancer-specific identification in the proteins, and pathways, were sought [5–7]. This is crucial since subclones harboring rare resistant mutations proliferate following decimation of sensitive cells [8]. These couple with gene rearrangements, fusions, and overexpression. All increase the number of active protein states [9]. Passenger mutations were observed to be unevenly distributed across cancer genomes, appearing to stem from regional chromatin accessibility [10,11]. Below, we briefly overview mutations in tumor signaling and metastases from our perspective.

Co-occurrence (or exclusivity) of two mutations in different genes (i.e., *in trans*) are a random event in cancer evolution. If two mutations in different genes occur less or more frequently than expected, they are mutually exclusive or co-occurring, respectively [12,13]. Mutual exclusivity was mostly observed in genes in the same or redundant pathways; co-occurrence was mostly observed in different, or parallel pathways [14]. In our definition, if the pathways recruit the same downstream protein families, they are redundant; if evolutionary-independent, they are parallel. Exclusivity has been attributed to the expectation that acquiring a second strong mutation in the same pathway is unsustainable for the cell since it risks its death [12,13]. It was proposed as associated with tumor subtype, synthetic lethality, and positive selection [15]. We clarified that co-occurrence is restricted [16,17], since a sustained, additive effect on signaling strength of same — or redundant— pathway driver mutations is likely to hyperactivate the proliferation signal, triggering an oncogene-induced senescence (OIS) cellular program [18–20].

OIS is a mechanism the cancer cell uses to proliferate- yet dodge cell death. Cancer cells opt for combinations of potent mutations. As early as 1953 [1], and again in 1969 [2], 1971 [3], 1999 [4], 2002 [5], 2015 [21], and more recently, it has been established that multiple co-existing mutations are required for the emergence of cancer. We also know that the number increases during proliferation. Yet, cells survive, suggesting that OIS combinations were sidestepped, or more likely, since somatic mutations emerge sporadically, cells harboring excluded combinations did not endure [22].

OIS, a tumor-suppressive mechanism arresting cell cycle progression, can be the main reason for excluding the co-occurrence of driver mutations in the same, here MAPK, pathway [23–25]. Irreversible senescence expresses strong oncogenic stimuli [26]. Overexpression of oncogenes such as *KRAS*, *BRAF*, and *MYC*, which also generate strong proliferation signal, induce OIS, as does increased PI3K/AKT signaling. Loss of *PTEN*, which negatively regulates the PI3K/AKT pathway, can trigger OIS through a p53-dependent pathway [27], or via coupling with strong PI3K mutational variants. Both constitutively generate signaling lipid PIP_3_. Sustained hyperactivation of the PI3K/AKT/mTORC1 pathway results in OIS [28,29]. Examples of mutually exclusive relations between gene pairs include *BRCA2*-*TP53*, *BRCA1*-*PARP1*, and *PTEN*-*PIK3CA* in breast cancer; and *BRCA1*-*CCNE1*, *BRAF*-*KRAS*, *ERBB2*-*KRAS* in ovary cancer [30]. Tumor suppressors can be advantageous and detrimental contingent upon the oncogenic context [31]. A third hit—within the same or a different gene in the pathway—may represent alternative routes to tumorigenesis [32].

The mechanisms underlying the mutual exclusivity of oncogenes may depend on the tumor type and interacting oncoproteins. *KRAS*^G12D^ and *BRAF*^V600E^ are mutually exclusive in lung cancer. Their co-expression triggers oncogene-induced senescence (OIS) [33]. Impaired B-Raf activation may avoid OIS, contributing to mutual exclusivity among certain *BRAF* mutations [34]. Activating mutations in RAS- and RAF-family proteins, especially the strong *BRAF*^*V600E*^ hotspot is one example [35,36]. Absence of co-occurring *NRAS* and *BRAF* variants, such as *BRAF*^*V600E*^, in the TCGA skin cutaneous melanoma cohort illustrates mutual exclusivity. Similarly, *BRAF* and RAS gene family mutations are mutually exclusive in metastatic colorectal cancer tumors [35].

Here we aim to discover (i) cancer-specific mutations that co-exist (*doublets* or *double mutations*) in different proteins in *tumor-specific* signaling, in primary tumors and in metastases, and (ii) those that are excluded. These are significant aims. For the first (i), knowledge of mutations that co-exist in different proteins and mapped onto pathways, can aid in knowledge-based combination drug strategy [37,38], and identify cancer metastases. For the second (ii) synthetic lethality, where a strong mutation in one gene product can be sustained but not in two, parallels excluded multiple strong drivers, clarifying its molecular basis. Related to (i), is the cardinal question of whether same tumor type cells which harbor common driver mutations are likely to adopt common drug resistance mechanisms.

Here we reveal rare but impactful mutation patterns across proteins that drive distinct phenotypes, informing oncology of drug combinations. We observe that mutations disrupting similar functions are mutually exclusive. As to metastatic tumors, some mutations *in trans* are significantly higher. We provide a list of actionable mutations for metastatic tumors in breast cancer cohort and a tree of double, triple, and quadruple co-existing patterns across the pan-cancer metastatic tumors. These also address the questions of *why these signatures*, and *what can they tell us about the possible molecular mechanisms which are involved*. As to synthetic lethality, which has been of broad interest due to its pharmacological potential [39–42], we provide a list, a rationale, and raise the possibility that OIS can provide the molecular basis of synthetic lethality.

## Results

### Tissue distribution and functional relevance of co-existing mutation pairs in cancer

**Fig 1:**
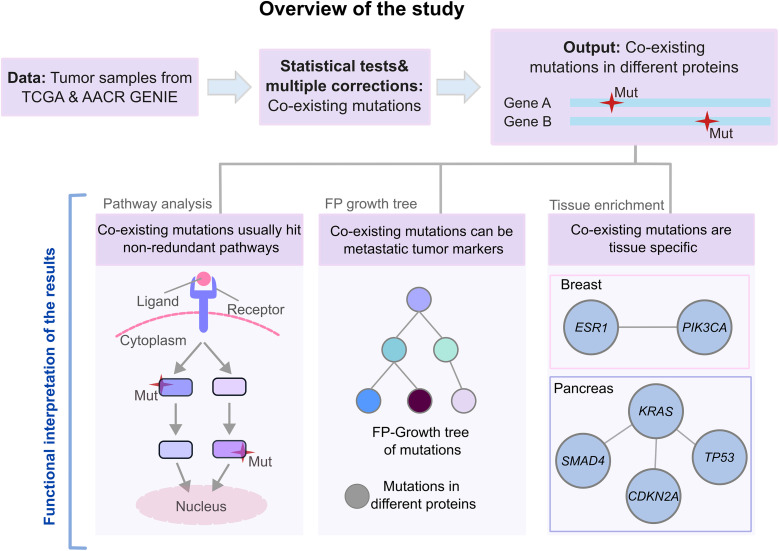
Overview of the study. We obtained tumor mutations from TCGA and AACR GENIE datasets. Then, we applied Fisher’s Exact Test followed by Benjamini-Hochberg multiple corrections. After obtaining 3424 significant mutation pairs, we mapped their components to pathways and tissues for functional interpretation. This helped identify co-altered pathways and protein pairs harboring the mutations specific to tissues. We also identified metastatic tumor markers by utilizing the Frequency Pattern (FP) Growth Tree algorithm.

To understand how mutations influence cancer evolution and how pre-existing mutations affect tumor initiation and metastasis [43], we analyzed statistically significant mutation pairs in different proteins using ~60,000 tumor samples from TCGA and GENIE pan-cancer datasets [44]. After identifying significant pairs, we mapped them to pathways and tissues and used Frequency Pattern Growth Tree analysis (treating each tumor as a transaction and alterations as items) to identify potential metastatic markers from co-existing mutations. Our analytical approach is outlined in Fig 1.

**Fig 2:**
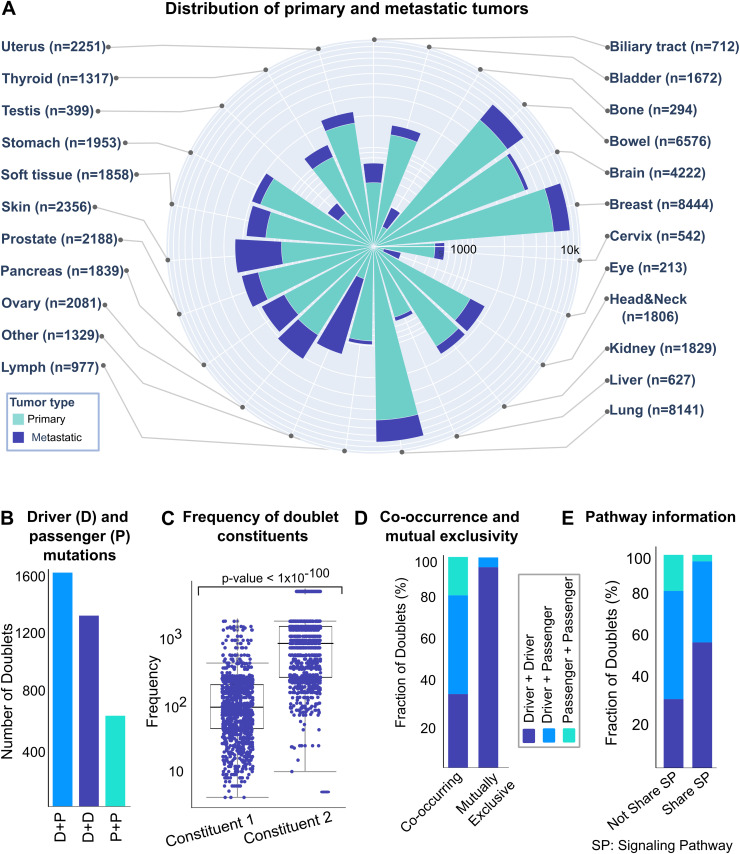
Statistics of mutation pairs in different proteins. **(A)** The windrose plot shows the number of primary (blue) and metastatic (light blue) tumors among tissues with at least 50 samples. The number of all tumors for each cancer type is specified in parenthesis. Breast, bowel, and lung have the highest number of primary tumors. Although the total number of metastatic tumors are less than primary tumors overall, skin, breast, intestine, lung, ovary, and prostate are among the relatively more metastatic. **(B)** There are 3424 significant mutation pairs in different gene (Fisher’s Exact Test p < 0.05, Benjamini-Hochberg q < 0.3). Their compositions are D + P (n = 1554), D + D (n = 1268) and P + P (n = 602) where D is “Driver” and P is “Passenger” mutation. **(C)** Box plot showing the frequency of mutation pairs constituents. For each pair, the component with lower frequency is put into “Constituent 1” and the other into “Constituent 2”. The figure shows that most pairs are composed of a frequent and a rare mutation. **(D)** Stacked bar plot of the mutation pairs in different proteins for 3307 co-existing and 117 excluded. For co-existing mutations, the distribution is as follows: D + D = 1155, D + P = 1550, P + P = 602. Excluded pairs include 113 D + D and 4 D + P. D (Driver), P (Passenger). **(E)** We call a protein pair A|B if there are mutations on proteins A and B. We divided the pairs into two groups: if the pair belongs to a common pathway, we put it into “Shared Signaling Pathway”, otherwise “Not Share Signaling Pathway”. 1284 protein pairs share at least one signaling pathway where the corresponding mutations are: 754 D + D, 480 D + P, 36 P + P). 638 of them do not share a signaling pathway, and 205 are D + D, 324 D + P, 108 P + P. For the remaining pairs, at least one of the proteins harboring them does not have pathway information. Co-existing mutations are usually present on protein pairs residing in different pathways and this could imply some driver mutations need a helper passenger mutation from a different pathway.

The distribution of metastatic tumors across tissues with at least 50 samples is shown in Fig 2A. Notably, skin, breast, intestine, lung, ovary, and prostate cancers include a relatively higher proportion of metastatic cases, although primary tumors still outnumber metastases in all tissues.

Analyzing missense mutations yielded 3,424 significant mutation pairs across 285 proteins (Fisher Exact Test p < 0.05, Benjamini-Hochberg q < 0.3) (S1 Table). Using the Catalog of Validated Oncogenic Mutations from the Cancer Genome Interpreter [45], mutations were labeled as drivers (D) or passengers (P). The pairs comprised 1554 D + P, 1268 D + D, and 602 P + P combinations (Fig 2B). We categorized mutation pairs by placing the lower frequency mutation as “Constituent 1” and the higher as “Constituent 2” (Fig 2C, and Fig A in S1 Text). Most pairs showed one frequent and one rare mutation. Of the total pairs, 3307 co-existed and 117 were excluded (Fig 2D, S1 Text). Co-existing pairs included 1155 D + D, 1550 D + P, and 602 P + P, while excluded pairs comprised 113 D + D and 4 D + P.

To assess whether mutually exclusive gene pairs in our analysis were enriched for synthetic lethal interactions, we compared our results with established synthetic lethality databases (SynLethDB and BioGRID [46,47]). We identified that 32% (12/37) of mutually exclusive pairs overlapped with known synthetic lethal interactions. Statistical analysis using Fisher’s exact test revealed significant enrichment (p-value = 9.33 x 10^-6^, OR = 6.32), indicating that mutual exclusivity strongly correlates with synthetic lethality. The overlapping pairs included prominent cancer-related genes such as *AKT1*|*PIK3CA*, *BRAF*|*KRAS*, and *EGFR*|*TP53*. In contrast, co-occurring gene pairs showed no significant enrichment for synthetic lethality (p-value = 0.895, OR = 0.54), suggesting that mutual exclusivity serves as a more reliable indicator of synthetic lethal relationships and may be valuable for identifying clinically relevant therapeutic targets.

To address whether mutually exclusive mutations potentially encoding oncogene-induced senescence (OIS), we conducted Gene Ontology enrichment analysis at MSigDB of the mutually exclusive mutation set. This analysis revealed significant overrepresentation of processes critical to cellular stress responses, including apoptotic processes (FDR q-value = 3.13 x 10^-10^), regulation of cell population proliferation (FDR q-value = 1.2e-9), and regulation of programmed cell death (FDR q-value = 7.04 x 10^-9^). Additional enrichment was observed in pathways involving phosphorus metabolic process regulation, protein modification processes, and phosphorylation regulation (FDR q-values between 7.38 x 10^-10^ and 3.31 x 10^-9^). These enriched terms reflect key hallmarks of oncogene-induced senescence, particularly the balance between proliferative signaling and growth arrest characterizing the senescent state, providing substantial evidence supporting our hypothesis that mutually exclusive mutations are enriched in pathways contributing to or responding to oncogene-induced stress [48,49].

Our analysis identified 3424 pairs with significant co-existing mutations (denoted as A|B when mutations occur in proteins A and B in the same tumors). Analysis of pathway relationships using the KEGG database (detailed methods in S1 Text) revealed 1280 protein pairs sharing at least one pathway (754 D + D, 487 D + P, 39 P + P), while 638 pairs (206 D + D, 324 D + P, 108 P + P) did not share pathways (Fig 2E). We identified 1,284 protein pairs containing 3,424 doublets and examined their tissue distribution in two categories: oncogene-oncogene (OG-OG) and oncogene-tumor suppressor (OG-TSG) pairs (detailed methods in S1 Text). Node colors show the fraction of samples with mutations in both proteins, normalized to samples with mutations in either protein per tissue. For clarity, we displayed OG-OG pairs with >10 mutation pairs (47 total) (Fig B in S1 Text) and OG-TSG pairs with >20 mutation pairs (54 total) (Panel B of Fig B in S1 Text).

The odds ratio analysis assessing whether the co-existing mutation constituents occur more (or less) frequently than expected under the null hypothesis of independence identified 3,307 co-existing mutation pairs. Notably, 45% of the significant co-occurring pairs involved a driver–passenger (D–P) combination, suggesting that passenger mutations may not arise entirely at random. Instead, they may be functionally tolerated or even selected in the presence of specific driver mutations, hinting at a possible cooperative or permissive relationship. In this context, the passenger mutation could contribute to pathway crosstalk, fine-tune signaling, or influence the tumor phenotype in a driver-dependent context. Our findings suggest a non-random pattern of co-occurrence, possibly indicating that certain passenger mutations are selectively maintained in the presence of specific drivers, potentially supporting tumor progression through complementary or compensatory mechanisms across pathways.

Most mutually exclusive pairs comprise driver mutations of varying frequencies, indicating strong oncogenic signals are complemented by less frequent mutations. *These patterns highlight the prognostic potential of mutation combinations* (detailed methods in S1 Text), and functional evaluation for therapeutics.

### Co-existing *KRAS* and *TP53* mutations alter transcriptional networks in pancreatic cancer

A driver alteration can influence protein signaling and transcriptional profiles. In transcriptional regulation networks, altered proteins may impact upstream activities via feedback loops [50]. We analyzed transcriptional differences between pancreatic adenocarcinomas (PAAD) with mutation pairs and those with single mutations.

*KRAS*^G12D^ is one of the most frequent mutations in pancreatic cancer, often paired with *TP53* mutations that impair DNA binding. Among 1502 PAAD tumors, 1007 harbor *KRAS*^G12D^ mutations. Of these, 397 tumors (from TCGA and ACCR GENIE) exhibit significant mutation pairs involving *KRAS*^G12D^ and *TP53*, including mutations at positions 248 and 273 (DNA contact sites) and position 175 (affecting Zinc ion binding, destabilizing p53). Group 1 includes 24 PAAD tumors with *KRAS*^G12D^-*TP53* significant mutation pairs, while Group 2 (71 tumors) contains single mutations in either *KRAS* or *TP53*, or mutations not contributing to significant pairs. Group1 and Group 2 contain tumors only from TCGA with the available transcriptomic data in PAAD.

**Fig 3:**
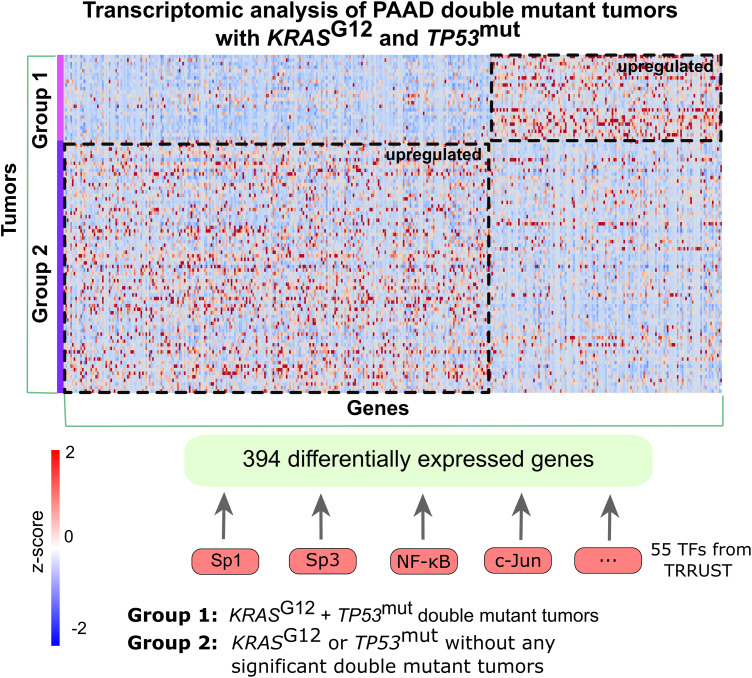
Transcriptome analysis of tumors with mutation pairs. Comparison of gene expression profiles of double mutant and single mutant *TP53* cases in *KRAS*^G12^ mutant PAAD tumors revealed 394 differentially expressed genes (137 upregulated and 257 downregulated) between Group 1 and Group 2. Group 1: PAAD tumors having at least one significant pair composed of *KRAS*^*G1*2^ and *TP53* mutations); Group 2: PAAD tumors having mutation either in *KRAS* or in *TP53* or mutation in any of these proteins that does not contribute to a significant pair. The heatmap shows the z-scores of differentially expressed genes (DEGs) across patient tumors. 55 TFs are retrieved as the transcription factors regulating the differentially expressed genes including the main regulators *SP1*, *SP3*, *NFKB1*, *JUN* and *TP53* obtained from TRRUST version 2 dataset, a manually curated database of human and mouse transcriptional regulatory networks. *SP1 (*encodes Sp1), *SP3* (encodes Sp3), *NFKB1*, *JUN* and *TP53* (encodes p53).

Transcriptomic analysis revealed 394 differentially expressed genes (Fig 3, 137 upregulated, 257 downregulated; adjusted p-value < 0.05, |log2FC| > 0.5) between Groups 1 and 2, with *p*-values corrected for multiple hypothesis testing using the Benjamini-Hochberg method. Group 1 tumors showed enrichment of proteins involved in immune response, cell proliferation, and cell-cell signaling. Using the TRRUST (v2) dataset, 55 upstream transcription factors (TFs) were identified (Fig 3), including *SP1 (*Sp1), *SP3* (Sp3), *NFKB1*, *JUN* and *TP53* (p53). A regulatory network constructed with CancerGeneNet highlighted upregulated proliferation phenotypes, aligning with the transcriptional impact of *TP53* driver mutations compared to single mutations [51,52].

Sp1 (Specificity Protein 1) is overexpressed in various tumor types and correlates with poor patient survival. High levels of Sp1, Sp3, and Sp4 contribute to cancer cell development, survival, and migration to tissues, including the pancreas [53,54]. In pancreatic ductal adenocarcinoma (PDAC), co-occurring *TP53* and *KRAS* mutations drive tumor growth and metastasis through interaction with CREB-1 (cAMP responsive element binding protein 1) [55]. Mutant p53 and CREB-1 upregulate FOXA1, a pro-metastatic pioneer transcription factor that activates its network and promotes WNT/β-catenin signaling, a key driver of PDAC metastasis. Inhibiting CREB-1 reduces *FOXA1* and β-catenin expression, limiting PDAC metastasis. Additionally, *TP53* missense mutations in *KRAS*-transformed PDAC are linked to poor differentiation and exhibit gain-of-function properties [56].

*Collectively, these show that co-existing KRAS and TP53 mutations alter transcriptional networks in pancreatic cancer, calling for reconsideration of PDAC drug regimen combinations accounting for both to restrain drug resistance*.

### Functionally similar alterations do not co-exist in tumors

**Fig 4:**
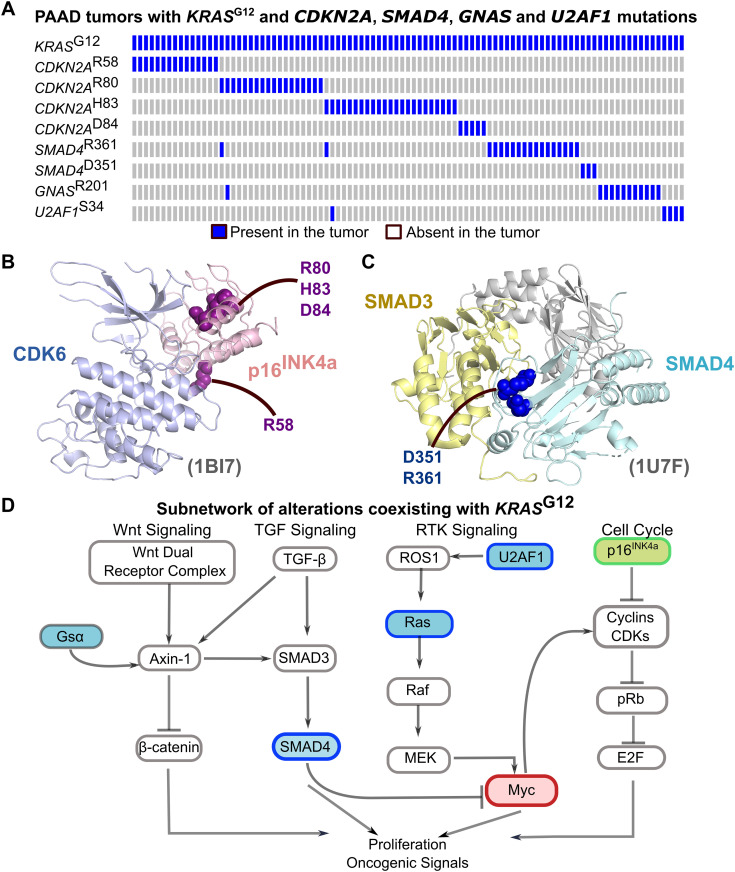
Co-existing mutations in PAAD tumors. **(A)** Oncoprint of the mutations that coexist with *KRAS*^G12^. *KRAS*^G12^ partners with *CDKN2A*, *SMAD4*, *GNAS*, and *U2AF1* which show a mutually exclusive pattern. **(B)** The structure of *CDKN2A*/*CDK6* complex shows that the mutations R58, R80, H83, and D84 are in the interface region between *CDKN2A* and *CDK6.* **(C)** *SMAD4* mutations D351 and R361 are in the interface between *SMAD4*/*SMAD3* complex. **(D)** A subnetwork of highly frequent alterations co-existing with *KRAS*^G12^ leading to proliferation and oncogenic signals.

We define alterations with similar phenotypic outcomes as “functionally equivalent,” which rarely or never co-occur and are often mutually exclusive. One example involves *KRAS*^G12D^ and *CDKN2A* deletions in pancreatic cancer. Mutually exclusive pairs may act in separate pathways but yield similar phenotypic effects. While co-existing mutations in *KRAS* are rare, they drive downstream changes promoting a proliferation phenotype. Fig 4A highlights mutations in *KRAS*^G12D^-mutated PAAD tumors, where alterations in *SMAD4*, *CDKN2A*, *U2AF1*, and *GNAS* are mutually exclusive and rarely co-occur. *CDKN2A* and *SMAD4* mutations, located in binding regions, rarely coexist in tumor proteins. *CDKN2A* mutations (R58*/Q, R80*, H83N/R/Y, and D84G) occur at the *CDK6* interface (PDB: 1BI7) (Fig 4B), with all but R58*/Q spatially clustered. These mutations disrupt *CDKN2A-CDK6* interactions, inhibiting cyclin D binding and retinoblastoma phosphorylation, thereby suppressing proliferation. *SMAD4* mutations at D351 and R361 localize to the SMAD4/SMAD3 heterodimer interface [57] (PDB: 1U7F (trimer)) (Fig 4C). These mutations disrupt *SMAD* homo- and hetero-oligomerization. All mutually exclusive mutations shown in Fig 4 are on non-overlapping paths linked to *SMAD4* (encodes Smad4) and their functional outcome may be similar. *U2AF1* and *GNAS* (encodes Gαs) paths are linked to Ras and downstream proliferation phenotype (Fig 4D).

Co-existence of *MYC* amplification and *CDKN2A* deletion is relatively rare. *MYC* drives proliferation, while *CDKN2A*, a tumor suppressor, restricts cell cycle progression in response to oncogenic or cellular stress. Their cooperation is linked to *TP53* mutations in the DNA-binding domain. This aligns with the absence of multiple strong alterations in the same or functionally similar pathways, as such combinations can trigger OIS [58,59].

*Altogether, such data pointing that functionally similar alterations do not co-exist in tumors, bespeak of which drug combination may be ineffective*.

### Distinct oncogenic signaling networks drive breast and pancreatic cancer subtypes

**Fig 5:**
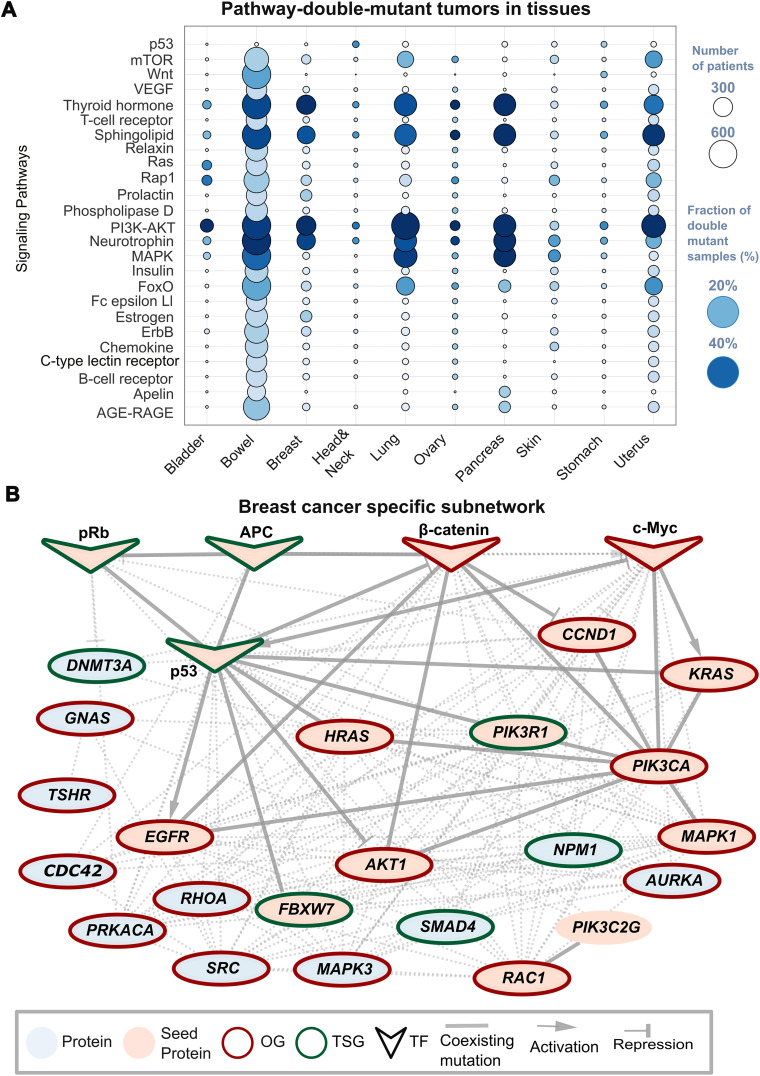
Breast cancer specific subnetwork. **(A)** Bubble plot representing the number and fraction of pathway-mutation pair- tumors in each tissue by node size and color, respectively. On the x-axis and y-axis there are 10 tissues and 25 pathways, respectively. Bowel and uterus have mutation pairs across all listed pathways while in breast, lung and pancreas tissues they accumulate in specific pathways including PI3K/AKT, MAPK, FoxO, Neurotrophin, mTOR. **(B)** Breast cancer specific subnetwork shows seed proteins, oncogenes and tumor suppressors obtained with the Page Rank algorithm from Omni Path with the seed proteins that have mutation pairs, and the tissue specific fraction is greater than 0.5. Blue nodes are the proteins from the PPI network, pink nodes are the seed proteins which are mutation pair components. Transcription factors (TFs) are V-shaped nodes. The border color is green if the gene is tumor suppressor gene and red if oncogene. The edges are solid lines if there is an edge between nodes in the PPI network that contribute to a mutation pair. Dashed lines depict the direct interaction in the PPI. Source and target shape are derived from TRRUST representing the activation or repression. TFs *RB1* (encodes the protein Rb1), *APC* (encodes the protein APC), *CTNNB1* (encodes Catenin Beta-1), *MYC* (encodes the protein Myc), *TP53* (encodes the protein p53).

The frequency of co-occurring mutations varies across tissue types, with affected proteins spanning multiple signaling pathways. We analyzed 3,424 significant mutation pairs, examining their tissue-specific distribution across 46 KEGG pathways (see S1 Text). Bubble plot (Fig 5A) illustrates the number and fraction of tumors with mutation-associated pathways across 10 tissues and 25 pathways. *Bowel and uterus tissues show mutation pairs across all pathways, while breast, lung, and pancreas tissues exhibit accumulation in specific pathways*, including PI3K/AKT, MAPK, FoxO, Neurotrophin, and mTOR. Mutation pairs in breast, lung, and pancreas tissues accumulating in specific pathways prompt the construction of cancer-specific subnetworks for breast and pancreatic tissues. Seed proteins with co-existing mutations were selected based on thresholds: breast (≥0.5%) and pancreas (≥1.5%).

From 8,512 breast and 1,853 pancreatic tumors, a directed PPI network with 20,029 nodes and 276,179 edges was derived from OmniPath [60] (see S1 Text). For breast cancer, 15 seed proteins were used, with initialization scores set to 1 for the PageRank algorithm (α = 0.85). The resulting subnetworks contained 59 nodes and 343 edges for breast (Fig 5B and Fig C in S1 Text) and 40 nodes and 267 edges for pancreas (Fig D in S1 Text). Transcription factors regulating subnetwork nodes were identified, along with their functional roles (activation or repression) to determine arrow types.

To evaluate the robustness of gene prioritization to the choice of the damping factor (α) in the personalized PageRank algorithm, we performed a sensitivity analysis across multiple α values (0.70, 0.75, 0.80, 0.90, and 0.95). Using the same tissue-specific seed genes and the OmniPath protein–protein interaction (PPI) network [60], we compared gene rankings generated with each α value to those obtained with α = 0.85 using Spearman rank correlation. The correlations were consistently high across all tested values, demonstrating strong agreement in rankings. For the pancreas-specific analysis, Spearman coefficients ranged from ρ = 0.9922 to ρ = 0.9997, while for the breast-specific analysis, they ranged from ρ = 0.9944 to ρ = 0.9998. These findings indicate that gene prioritizations are highly robust to variations in the damping factor, supporting the reliability of results obtained with α = 0.85.

For breast cancer, the resulting subnetwork includes the TFs *RB1* (encodes the protein Rb1), *APC* (encodes the protein APC), *CTNNB1* (encodes Catenin Beta-1), *MYC* (encodes the protein Myc), *TP53* (encodes the protein p53). Oncogenes or tumor suppressor proteins (nodes) have red and green borders, respectively. Gene set enrichment analysis in MsigDB [61,62] and Webgestalt [63] yielded subnetwork proteins enriched in the following pathways prominently (panels A and B of Fig E in S1 Text): GPCR Signaling (41 proteins overlapped), G-alpha signaling events (28 genes), Chemokine signaling pathway (25 genes), GnRH signaling pathway (21 genes), Gap junction (20 genes), Endothelins (19 genes), LPA receptor mediated events (19 genes). GPCRs are crucial in tumorigenesis, angiogenesis, and metastasis by modulating heterotrimeric G-proteins pathways [64]. GnRH agonists inhibit cancer cell growth and invasion while suppressing ovarian steroid production [65,66]. Chemokines activate pathways like PI3K/AKT/NF-κB and MAPK/ERK, promoting proliferation [67,68]. Targeting these pathways offers potential for breast cancer treatments [69]. In the pancreas cancer subnetwork, key TFS such as *APC* (encodes APC), *RB1* (encodes pRb), *DNMT3A*, *TP53* (p53), *MYC*, *CTNNB1* (Beta Catenin-1) and *SMAD4* (Smad4) contribute to mutation pairs. Gene set enrichment analysis with WebGestalt (Panels A and B of Fig F in in S1 Text) revealed upregulated pathways like PI3K/AKT/NF-κB and MAPK/ERK signaling, and platelet activation.

To contextualize our results and distinguish biologically meaningful signals from random network effects, we examined pathways enriched in fewer than 50% of subnetworks reconstructed from randomized interactomes in breast and pancreatic cancers. These infrequently enriched pathways may represent more robust, non-random features of the original network topology. Notably, pathways such as IL-17 signaling, Hedgehog signaling, and Necroptosis—known to regulate inflammation, cell death, and immune evasion—appeared in only 39–44% of random subnetworks, suggesting genuine biological relevance in breast cancer subnetworks. In contrast, pathways like Autoimmune thyroid disease, Phagosome, and systemic lupus erythematosus were rarely enriched (≤10%), and several—including Olfactory transduction and Lysosome—were never enriched in the random subnetworks.

Pathways such as GPCR signaling, GnRH signaling, and chemokine-mediated cascades, while repeatedly enriched, have broad and pleiotropic roles in cellular signaling and may dominate enrichment outputs due to their general connectivity in the interactome. Despite this, the consistent enrichment of key oncogenic pathways—such as PI3K/AKT and MAPK signaling—in both the original and randomized settings support their centrality in cancer progression and aligns with the transcription factor disruptions identified in the pancreatic cancer subnetwork. While these pathways appear most prominent in terms of overlap size, their recurrence in analyses of randomized networks suggests that they may arise due to inherent network topology rather than specific biological signal.

In the pancreas cancer subnetwork, several signaling pathways—including IL-17, Adipocytokine, and Hedgehog signaling—were each enriched in 40% of subnetworks, while Pertussis signaling, involving GPCR activation, appeared in 44%. These pathways are known to influence inflammation, metabolism, and developmental signaling, all central to tumor biology. Oocyte meiosis (25%) may reflect dysregulation in cells. Their selective presence in the original network, but low recurrence across randomized versions, suggests these enrichments reflect disease-specific biological features.

Complex biological functions are mostly managed through protein-protein interactions (PPIs), and mutations occurring in proteins may alter the cell phenotypes [70]. Alterations perturbing PPIs can impact drug outcomes [71–73]. Detection of druggable oncoPPIs [71] is promising since some could constitute more cancer-specific therapeutic targets [74,75], although the pre-existing mutation load can impact the efficacy [8]. Among the Fujian cohort mutations classified as metastatic markers in gastric carcinomas, one is a *PTPRT* mutation [76], and another, Chondrosarcoma *TERT* promoter mutation is a metastasis marker [77]. A real-world clinicogenomic dataset permitted the discovery of biomarkers that predict treatment outcomes that affect patients’ survival [78]. Treatment-specific genetic alterations in non-small cell lung cancer (NSCLC) include mutually exclusive and co-occurring mutations [79,80]. A whole-genome sequencing of a large Swedish cohort study [81] aimed to identify prognostically relevant co-occurring mutations in microsatellite stable colorectal cancers. The analysis revealed 23 significant co-mutations, with specific pairs such as *APC*-*TCF7L2* and *BRAF*^V600E^-*RNF43* showing distinct associations with favorable or poor survival outcomes, highlighting the added prognostic value of co-mutation profiling over single-gene analysis.

Overall, coupled with the literature, our analysis indicates that pathways carrying co-existing mutations can help in fathoming the evolution of certain cancer types and guide target selection.

### Co-existing mutations are mostly in primary tumors and some rare mutations are signatures of breast cancer metastatic tumors

Genomic comparisons between primary and metastatic tumors show only moderate increases in mutation burden, with common mutations in TP53, *MYC*, and *CDKN2A* [82–85]. While these studies reveal genomic similarities, key questions remain about the molecular mechanisms driving metastatic phenotypes and their activation [86,87]. Analysis of 40,979 tumors across 25 cancer types found no distinct mutational patterns associated with metastasis [85].

**Fig 6:**
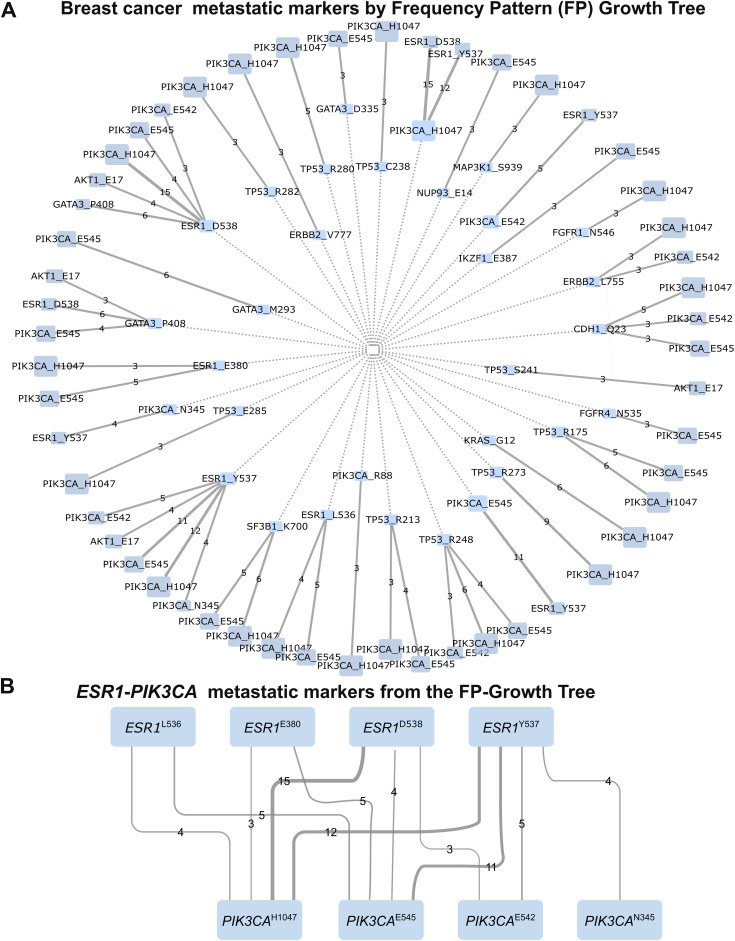
Rare mutations among the co-existing mutations are specific to metastatic tumors. **(A)** Frequency pattern growth tree of missense mutations shows breast cancer metastatic markers where links represent association between mutations. Edges represent correlation strength. Ancestral mutations are shown as dark blue nodes, leading to consequential light blue mutations. Mutation frequencies are represented by node sizes. Mutation pairs accommodated by *ESR1*, *GATA3*, and *PIK3CA* proteins are enriched in metastatic tumors. 45 mutation pairs from 31 nodes and 51 edges in the FP growth tree are among those evaluated as metastatic markers. **(B)** *ESR1*|*PIK3CA* mutations are enriched in breast cancer metastatic tumors derived from FP Growth Tree in panel A. *ESR1* mutations at positions 380, 536, 537 and 538 are paired with the major drivers of *PIK3CA* at positions 542, 545, and 1047.

**Fig 7:**
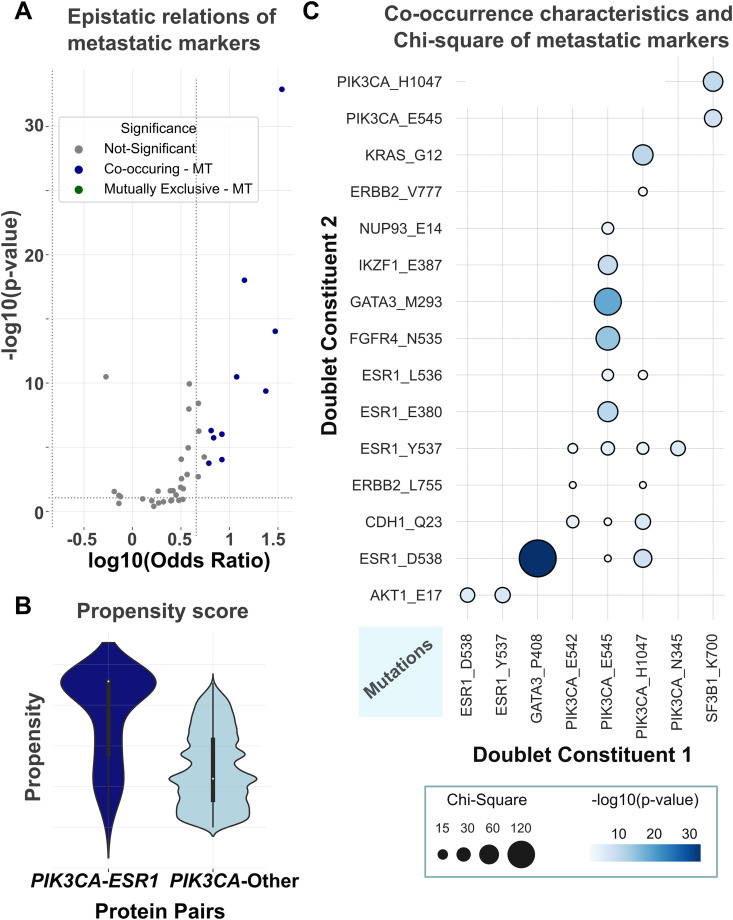
Metastatic marker statistics. **(A)** We visualize each alteration pair with a volcano plot, where the x-axis represents $\log_{10}(OR)$ and the y-axis represents $\log_{10}(Fisherp-value)$. Pairs with $\left|\log_{10}(OR)\right|>0.75$ indicate strong effect sizes, and a threshold of ${-log}_{10}(p)>1.65$ corresponds to p < 0.05, our chosen significance level as this threshold is widely used in biological research to denote statistical significance. **(B)** Propensity of mutation pairs from *ESR1*|*PIK3CA* and from other proteins where one side is from *ESR1* or *PIK3CA* in metastatic tumors. **(C)** The bubble plot evaluating all possible relationships of the mutations at the FP growth tree, where node color depicts coexistence characteristics, and node radius is proportional to the Chi-Square value of the same cohort.

Analysis of the AACR GENIE cohort’s primary and metastatic breast cancer samples revealed that while over 70% of mutation pairs exist in primary tumors, some pairs are metastasis specific. Using frequency pattern growth tree analysis (Fig 6), we examined mutation patterns in the Metastatic Breast Cohort (n = 2385), comprising 52% IDC, 25% BRCA, 9% ILC, 4.5% BRCNOS, and other subtypes. The FP growth tree, analyzing 456 mutations across 2385 samples, yielded 31 nodes and 51 edges, with 40 significant mutation pairs serving as potential metastatic markers (Fig 6A, S2 Table). Strong associations emerged between mutations in *ESR1*, *GATA3*, and *PIK3CA* in metastatic tumors, particularly *ESR1* mutations at positions 380, 536, 537, and 538 pairing with *PIK3CA* mutations at positions 542, 545, and 1047 (Fig 6B). A volcano plot analysis (Fig 7A) illustrated co-existence and mutual exclusivity patterns of metastatic markers, while further analysis confirmed *ESR1*|*PIK3CA* mutation pairs as significant metastatic indicators (Fig 7B, p-value < 10^-13^). *GATA3* mutations at positions 293, 335, and 408 showed coupling with *ESR1* or *PIK3CA* mutations, consistent with *GATA3*’s known functional relationship with *ESR1* in transcriptional regulation [88]. The relationships between mutations were visualized in a bubble plot (Fig 7C), excluding 330 pairs with fewer than three co-existing mutations. Analysis of 393 metastatic breast tumors from the Hartwig Medical Foundation (HMF) dataset [89] reveals that 22 tumors harboring both *PIK3CA* and *ESR1* mutations. Specifically, *PIK3CA* mutations at positions 542, 545, and H1047 paired with *ESR1* mutations at positions 380, 536, 537, and 538, confirming the metastatic markers identified by the FP growth tree algorithm.

In addition to these, we provide a tree of double, triple, and quadruple co-existing patterns across the pan-cancer metastatic tumors (Panels A and B of Fig G in S1 Text). To construct the tree in Panel B of Fig G, a minimum support of 7 × 10 ⁻ ⁵ and a minimum confidence threshold of 0.95 were used. In the tree, the node *TP53*^R273^ is followed by either one of the mutations on ATRX at the positions 1045, 1049, and 1426 where the third node of the triplet is *IDH1*^R132^. *IDH1*, *TP53*, and *ATRX* mutations are associated with low-grade astrocytomas collaborate to inhibit *SOX2* (a nucleosome-binding pioneer TF acting to make genes accessible to the transcription machinery) and prevent the development of human neural stem cells [90,91]. Quadruples include mutations from *BRAF, MSH3, BLM, RNF43, PLK2, NBN.* In response to the context-dependent nature of cancer genomics, we derived a breast cancer–specific co-mutation tree illustrating doublet, and triplet mutation patterns, rather than performing a pan-cancer analysis. Due to the limited sample size of the metastatic breast cancer cohort, we applied less stringent thresholds (minimum support = 0.0007, minimum threshold = 0.01). The triplets in metastatic breast cancer include *ESR1*^D538^+*PIK3CA*^H1047^ + *SF3B1*^K700^ and *PIK3CA*^E545^ + *GATA3*^M293^+*NUP93*^E14^. To improve interpretability, direct mutation pairs connected only to the null root were excluded. The resulting tree is shown in Fig H in S1 Text, and the corresponding data is provided in S3 Table.

As breast cancer represents 25% of female cancers globally, with hormone receptor-positive being the most prevalent subtype, marked by estrogen receptor (ER) expression. While endocrine therapy targeting ER is standard, resistance often develops through *ESR1* mutations, affecting up to 36% of metastatic breast cancer (MBC) patients [92]. Studies by Gerratana et al. revealed that *ESR1* codon 537 variants associated with ER and RAF pathway SNVs, *MYC* CNVs, and bone metastases, while codon 538 variants correlate with cell cycle SNVs and liver metastases. *PIK3CA* codons 1047 and 542 showed associations with CNVs, informing therapeutic strategies [93].

*Thus, although primary and metastatic tumors share driver mutations* [85]*, most are in primary tumors. Some rare mutations appear enriched in metastatic tumors and may serve as potential markers, although further validation with larger datasets is required to establish their significance as metastatic signatures. A more comprehensive cross-cancer metastases analysis is vital for early detection.*

## Discussion

Tumorigenesis involves complex protein alterations [94–97], where co-existing mutations in different proteins (*in trans*) undergo evolutionary selection [12,13]. While mutations in parallel pathways often co-exist, those in identical or redundant pathways typically show mutual exclusion, reflecting cellular sustainability limits [14]. This exclusion pattern links synthetic lethality and oncogene-induced senescence (OIS) [15–20,98], where hyperactivation of proliferation signals becomes unsustainable.

Understanding cancer evolution requires systematic investigation of mutation epistasis [15,98,99–102], supported by both driver-passenger mutation classification tools [30] and methods for identifying mutually exclusive gene sets [103]. There are several tools for identifying biologically meaningful, mutually exclusive gene sets [30], including statistical frameworks (MEScan [103], MEGSA [104], CoMET [105]) and driver module detection (MEMo [106], CoMDP [107]). These approaches, ranging from statistical frameworks to driver module detection [104–108], culminate in tools like SELECT [108] that capture evolutionarily dependent alterations affecting drug response. Our analysis of *in trans* mutations identified 3424 significant co-existing mutations, predominantly comprising one rare and one frequent mutation, with 3307 co-existing pairs and the remainder being mutually exclusive. Co-existing mutations can enhance oncogenic signaling, while mutual exclusivity may result from unsustainable hyperactivity through same-pathway mutations or combinations with pioneer transcription factors like *FOXA1* [109]. The FP-growth tree method [110] revealed increased frequency of *PIK3CA* and *ESR1* mutation pairs in metastatic versus primary tumors.

Previous research on *in cis* mutations (multiple mutations in the same proteins) [44,51,111–113] showed increased oncogenic activity and drug sensitivity, with double mutations enhancing PI3K signaling and tumor growth. Clinical trials suggest double-mutant breast cancers respond better to PI3K inhibitors than single-mutant cases [111], though exceptions exist, as seen in *EGFR*^L858/T790^’s increased resistance compared to *EGFR*^L858^. Building on this, studies of PI3Kα variants showed that co-existing mutations—often combining a hotspot with a weaker mutation—can shift conformational ensembles toward more active states and expose mutation-specific cryptic pockets, underscoring the need for variant-specific combination allosteric therapies [114].

Molecular studies have advanced our understanding of metastatic mechanisms and prognostic factors in cancers, particularly focusing on mutation patterns and their clinical implications. Wang et al. analyzed 114 Chinese NSCLC patients, finding higher metastasis in older patients with TP53 mutations, while *EGFR*, *ASXL2*, and *STK11* mutations correlated with better survival [115]. Studies on thyroid cancer highlighted advances in biomarker detection enabling personalized treatment [116]. Analysis of 154 lung cancer brain metastases revealed greater tumor heterogeneity than primary tumors, with shared mutations (*TTN*, *TP53*), enhanced mitochondrial metabolism but suppressed immunity; combining mitochondrial-targeting drugs with immunotherapy improved mouse survival [117]. In 1817 *KRAS*-mutant lung adenocarcinomas, *KEAP1* and *SMARCA4* mutations correlated with metastasis and poor survival, while *STK11*’s impact varied by metastatic site and *KEAP1* status [118]. Potential prognostic biomarkers in lung adenocarcinoma were identified through integrative analysis of mRNAs, miRNAs, and lncRNAs and cell cycle–related ceRNA network [119].

A limitation of our approach is the lack of redundancy reduction by tissue type, which may have allowed cancers with larger sample sizes—such as breast and pancreas—to dominate some signals. While this enabled pan-cancer discovery, it may introduce tissue bias. We later addressed this by identifying the tissue distribution of key doublets and selecting case studies from cancer types with both enrichment and sufficient sample size. Yet another limitation of our approach is the reliance on KEGG as pathway database; while KEGG is widely used and well-curated, different pathway resources such as Reactome [120] or WikiPathways [121] may yield alternative pathway overlaps due to variations in pathway definitions and gene assignments.

This study addresses two critical yet understudied challenges in cancer research: (i) proposing candidate markers potentially linked to metastatis; (ii) mutations whose combinations may be linked to oncogene-induced senescence (OIS), thus are rare, or absent in cancer genomes. As to point (ii), we conjecture that the absence of these combinations may be because the oncogenic signal they generate is too strong for the cell to tolerate. Mutations related to OIS likely emerge sporadically during cancer evolution, and further work is needed to validate this hypothesis. Cells do not have a mechanism for *a priori* negative selection. Rather, negative selection, or exclusion, is the outcome of cell death. Here we provide a list, a rationale, and suggest that OIS can provide the molecular basis to synthetic lethality, which has been investigated on the gene level. As to (i), to our knowledge, we provide a first candidate list of metastatic markers specific to metastatic samples, including actionable mutations for metastatic tumors, as well as a tree of double, triple, and quadruple co-occurrence patterns across the pan-cancer metastatic. This list (S2 Table) may help in early metastases detection. Advanced computational approaches allow detection of patterns. Co-existing mutation signatures for metastatic tumors, coupled with identification of the respective proteins and pathways, can create interactive maps. Linking them with drugs can create immensely useful tools for the attending physicians [122].

Finally, while the usefulness of metastatic markers to drug discovery is self-evident, exactly how to identify OIS mutations in protein pairs is less so, as indeed is also the case in synthetic lethality [123]. OIS restriction is observed in same- and redundant-pathways. Classically, drugs aim to block protein activity and downstream signaling. Introducing drugs that recreate their action is expected to lead to cell death. At the same time, controlling the signal is challenging, as strong signals can be associated with cell proliferation, but too strong a signal may result in oncogene-induced senescence.

## Materials and methods

### Data collection and processing

The data and preprocessing protocol is same as in [44] and the corresponding code and input data files are available at https://github.com/bengiruken.

### Identification of significant co-existing mutations

Out of a total of 395,801 mutations with a variant allele frequency (VAF) greater than 0.125 [44], 20,157 mutations were found in at least three non-hypermutated tumor samples. We generated all possible binary combinations of these 20,157 mutations, excluding pairs from the same gene. For each unique mutation pair, we created a contingency table. This process yielded 366,573 potential co-existing mutations in different genes, which we evaluated across 59,048 non-hypermutated tumor samples using Fisher’s Exact Test for statistical significance where the contingency table is as follows (Table 1):

**Table 1:** Contingency table.

a (tumors with both mutations)	b (has only first mutation)
c (has only second mutation)	d (has neither of the mutations)

where *d* = 59,048 − (a + b + c) and 59,048 is the number of non-hypermutated samples. We did not apply any initial filter based on the number of tumors with co-existing mutations—cases where *a* = 0 were also tested. We then adjusted for multiple comparisons using the Benjamini-Hochberg method and proceeded with downstream analyses using 3,424 mutation pairs that had a q-value below 0.3 and were observed in at least three tumor samples. If a co-existing mutation constituent is among the 5601 driver mutations from the Catalog of Validated Oncogenic Mutations (Cancer Genome Interpreter [45]), it is labeled as known driver (D), otherwise passenger (P).

### Transcriptome analysis

To identify differentially expressed genes in the group of patients with co-existing mutations compared to the single mutant patient group, we downloaded RNA-seq transcriptome data of the TCGA project from the cBioPortal database (https://www.cbioportal.org). We used median Transcripts Per Kilobase Million (TPM) values of RNA-seq data of PAAD cohort of TCGA. For the PAAD cohort, 177 patients with TPM values, we constructed two groups, where Group 1 is tumors having at least one significant co-existing mutation of type *KRAS*^G12D/V/C ^+ *TP53*^mutation^ and Group 2 is tumors having either single mutant *KRAS*^G12D/V/C^ or single mutant *TP53*. We calculated the log_2_FC value of each gene between the double mutant and single mutant groups by using the formula:


$$log_{2}FC=\frac{Mean\left(Gene^{\prime }s\;TPM\;values\;among\;Double\;Group\;Patients\right)}{Mean(Gene^{\prime }s\;TPM\;values\;among\;Single\;Group\;Patients)}$$ (1)

We identified differentially expressed genes between the double mutant tumors group and the single mutant tumors group (comparison of means of TPM values by Mann-Whitney U-Test). If |$log_{2}FC$| > 0.5 and adjusted p-value < 0.05 we considered the corresponding gene as differentially upregulated or downregulated in the double mutant group. Benjamini-Hochberg procedure to control the false discovery rate (FDR) for multiple correction was performed using the multipletests function in the statsmodels Python package. We continued our analysis with these differentially expressed genes, and calculated z-scores of each gene as follows:


$$z=\frac{TPMvalueofGene-\mu }{\sigma }$$ (2)

where is µ is the mean of TPM values and σ is the standard deviation across all samples in the double mutant and the single mutant groups. After obtaining z-scores for each gene, we sorted the genes as downregulated and upregulated and represented these values as a heatmap (https://seaborn.pydata.org).

We used Webgestalt (http://www.webgestalt.org) for the gene set enrichment analysis where the functional database is selected from Reactome and significantly up- or down-regulated pathways are found. FDR threshold is selected 0.05 and the list of genes ranked by their logFC values are given as input.

### Personalized PageRank algorithm

We used a network diffusion-based algorithm to find the most affected region of the interactome given a set of nodes. Given a directed or undirected graph G(v,e) where v ∈ V and e ∈ E and a set of seed nodes S ⊆ V, the personalized PageRank algorithm solves the seed set expansion problem, where it finds which additional nodes may exist in the community besides the nodes in S and ranks them according to their importance. We used the PageRank [124] function implemented in Python networkx library [125]. The damping parameter, alpha, is selected as 0.85 and the number of iterations are 100.

We applied the personalized PageRank algorithm to prioritize genes based on their proximity to tissue-specific seed genes within the OmniPath PPI network [60]. Following standard practice in network analyses and the original PageRank formulation [126], we set the damping factor α to 0.85, which balances local and global network exploration. To assess sensitivity to this parameter, we recomputed PageRank scores using α values of 0.70, 0.75, 0.80, 0.90, and 0.95, keeping the network and seed genes constant. We then compared gene rankings derived at each α to the rankings obtained with α = 0.85 using Spearman rank correlation, a non-parametric statistic appropriate for evaluating rank-order consistency.

### Tissue specific subnetworks

To determine tissue specific seed proteins to perform Personalized PageRank algorithm for obtaining tissue specific subnetworks, we calculated the fraction of double-mutant tumors among all tumors in the corresponding tissue for each doublet. Then we evaluated the gene couples harboring co-existing mutation components as seed proteins if the fraction of the tumors with co-existing mutations is greater than 25%. We assigned initial weights as 1. We run the PageRank algorithm on the PPI network from OmniPath [60] with alpha = 0.85. We added the edges with score greater than the threshold 0.001 to the breast specific subnetworks.

We evaluated 46 signaling pathways from KEGG. We obtained pathway-double-mutant tumors by taking the union of all double mutants when at least one of the proteins belong to the corresponding pathway. We filtered the pathways that have less than 80 double mutant tumors and tissues and pathways that have less than 10% double mutant tumor rate. We constructed cancer type specific subnetworks for breast and pancreas tissues with seed proteins that have co-existing mutations and tissue specific double mutant fraction is greater than a certain threshold (thresholds are as follows: breast, 0.5; pancreas, 1.5). The number of seed proteins for breast cancer and pancreas cancer are 12 and 23, respectively.

Shared pathway membership between genes was determined based on overlap in KEGG gene sets, not by matching pathway names, to ensure functional relevance rather than semantic similarity.

To evaluate the robustness of pathway enrichment results against the native topology of the PPI network, we generated 100 randomized directed networks using a degree-preserving edge-swapping algorithm. For each randomized network, PageRank scores were computed using a damping factor of 0.85 and a fixed personalization vector. Genes exceeding a threshold score of 0.002 were used to reconstruct subnetworks.

We then performed pathway enrichment using the KEGG 2021 Human gene sets in gmt format with the GSEApy [127] package. A local Enrichr-based enrichment approach with retry mechanisms was used to ensure reliable results. For each pathway enriched in the original subnetwork, its frequency across the 100 randomized subnetworks was calculated and expressed as a percentage. This allowed identification of pathways that were specifically enriched in the original network and not due to network structural properties.

### FP Growth Tree construction

We used the mlextend library’s FreqItems and AssociationRules functions for mining frequent item sets and association rules. The FP growth algorithm is selected for tree construction where each node represents one alteration and each edge in the tree represents the association of the nodes [110]. In the constructed tree, all nodes in the path from root to the distant node are associated with each other and strongly present together in the tumors. The implementation of this workflow or additional documentation on how the FP-Tree algorithm is adapted for this specific use case is available at https://github.com/ugur0sahin/FMPSeeker.

The tendency of the alterations to be specific to metastatic tumor is calculated by


$$propensity(i)\;=\;\frac{x_{i}/N_{i}}{X/N}$$ (3)

where *x*_*i*_ is the number of metastatic tumors having co-existing mutation *i*, *N*_*i*_ is the number of tumors having co-existing mutation *i*, X is the number of metastatic tumors and N is the number tumors in our dataset.

To assess co-occurrence among alterations in the metastatic breast cancer cohort, we first restricted our alteration matrix to this subset of patients. We then evaluated all unique pairs of alterations using itertools.combinations() in Python.

For each alteration pair (A, B), we extracted binary presence/absence vectors across samples from a pandas DataFrame and constructed a 2 × 2 contingency table based on the four possible joint states:

- **a**: both alterations present,
- **b**: only alteration A present,
- **c**: only alteration B present,
- **d**: neither present.

These counts form the basis for statistical testing. We used scipy.stats.fisher_exact to compute both the odds ratio (OR), calculated as


$$OR=\frac{ad}{bc}$$

and the Fisher exact test p-value for each pair. Additionally, we applied scipy.stats.chi2_contingency to compute Pearson’s chi-squared statistic and corresponding p-value.

**Note:** We present further details of the downstream analyses of co-existing mutations in the supplemental information S1 Text.

## Supporting information

S1 Text Contains supplemental text, methods and figures. (DOCX)

S1 Table Statistics of co-existing mutations on different proteins. (XLSX)

S2 Table Metastatic breast cancer co-existing mutation markers. (XLSX)

S3 Table Metastatic breast cancer triplet mutations in FP Growth tree. (XLSX)
