# Supplementary material for: Mutations in tumor signaling, metastases, and synthetic lethality establish distinct patterns
Source: PLoS Comput Biol. 2025 Aug 4;21(8):e1013351. doi: 10.1371/journal.pcbi.1013351 (PMC12342271; doi:10.1371/journal.pcbi.1013351)
Supplement: S1 Text — (DOCX) [file pcbi.1013351.s001.docx]

**S1 Text**

Pathway Analysis

We used 46 signaling pathways from KEGG: Kyoto Encyclopedia of Genes and Genomes [1] to associate with double mutant genes. We downloaded the relevant data from <https://maayanlab.cloud/Enrichr/>.

Oncoprint Maps

To reveal mutual exclusivity and co-occurrence patterns between double mutations we plotted oncoprint maps by using ComplexHeatmap [2] package of R and cBioPortal (<https://www.cbioportal.org>).

**Steps in building the FP growth tree**

***Built***

Workflow for analyzing mutation patterns in a dataset, specifically BreastMetastatic cohort, using frequent pattern mining, which is a common task in bioinformatics and data mining. This process appears to use the **mlextend** library's **FreqItems** and **AssociationRules** functions, which are likely Python implementations for mining frequent itemsets and association rules, respectively. Here's a more detailed explanation based on the FP-Tree (Frequent Pattern Tree) algorithm and the **mlextend** library:

***Input Data***

The workflow begins with a binary alteration matrix from the BreastMetastatic cohort. This matrix likely has cases as rows and mutations as columns, as binary format indicates the lacking/having of mutations corresponds with 0/1.

***Frequent Itemset Mining***

The **mlextend.FreqItems** function processes the binary matrix to find frequent mutation patterns. The function is parameterized by **minSupport** set to **0.001** (for Pan Cancer Tree in Panel B of Fig G), which is the minimum frequency a pattern must have to be considered **frequent**.

This process identifies sets of mutations that commonly occur together in the dataset. The frequent itemsets are parsed from the NULL tree, which is a reference to the initial state of the FP-Tree where no patterns have yet been identified.

***Association Rule Mining***

The frequent itemsets obtained are used to generate association rules with the **AssociationRules** function from **mlextend**.

This function looks for mutations that are highly associated with each other, based on a **minTreshold** parameter. This threshold determines how strong the association needs to be for a rule to be considered. It's noted that unlike a p-value, the confidence of these rules is inversely related to the p-value from statistical tests.

***Tree Construction***

Based on the association rules, new branches (paths) are generated and added to the NULL (ROOT) node of the FP-Tree. Only branches representing associated pairs are added.

This step constructs a tree where the paths represent the common mutations between different cases, revealing how certain mutations (ancestors) are associated with other mutations (descendants) within the dataset.

***Tree Visualization***

The layout and theme of the tree are adjusted according to the characteristics of the pairs/branches:

- The **EdgeName** is labeled with the **PairCounts**, which likely indicates the number of times that pair of mutations has been found associated.
- The **NodeSize** represents the frequency of the mutation or alteration, which could be visualized by larger nodes indicating more frequent mutations.
- The **EdgeWidth** is determined by the **PairCounts**, where a wider edge indicates a more frequently occurring pair of mutations.
- The **EdgeStyle** (dashed or linear) differentiates between mutation pairs (AlterationPair) and edges that stem directly from the root of the tree.
- The **ColorTones** used in the visualization distinguish between ancestors (*ANCE*, dark tones) and descendants (*CONS*, light tones) to show the directionality of associations.
- The GitHub link provided -> https://github.com/ugur0sahin/FMPSeeker

contain the implementation of this workflow or additional documentation on how the FP-Tree algorithm is adapted for this specific use case.

FP-Tree algorithm to find frequent patterns and strong association rules in a binary matrix representing mutation data from breast cancer metastatic cases. The final goal is to construct a tree visualization that meaningfully represents the relationships between different mutations, providing insights into the underlying patterns of mutation co-occurrence in the data.

***Workflow Diagram:***

***
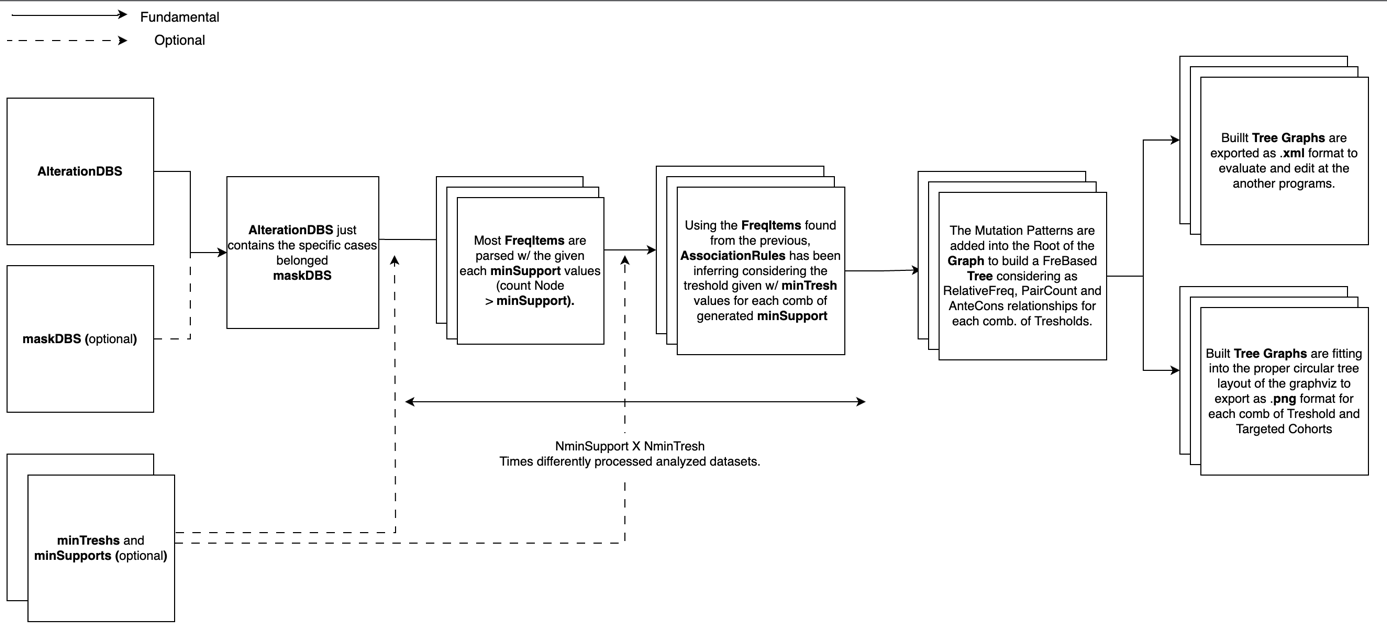
***

**Transcriptional analysis of PAAD tumors**

One of the most frequent mutations in pancreatic cancer patients is *KRAS*^G12D^. It pairs with a mutation in *TP53*, which impairs DNA binding. In the pan-cancer dataset, there are 1502 PAAD (Pancreatic adenocarcinoma) tumors, 1007 of these have *KRAS*^G12D^ mutations. There are 67 significant mutations pairing with *KRAS*^G12^ and at least one in TP53 in 397 patient tumors. Among them, mutations at positions 248 and 273 are directly in contact with DNA. Position 175 is far from the DNA binding region however it is in contact with a Zinc ion and a mutation at that position destabilizes p53, preventing DNA binding. Some tumors have *TP53* and *KRAS*^G12D^ mutational pairs. Because this coupling likely impacts transcriptional regulation, we compared the transcriptome profiles of Group 1 (defined as PAAD tumors having at least one significant *KRAS^G1^*^2D^ and *TP53* mutation pairs) to Group 2 (PAAD tumors having mutation either in *KRAS* or in *TP53* or mutation in any of these proteins that does not contribute to a significant pair) (Figure 3). Group 1 has 24 patient tumors. Group 2 has 71, in 35 of which TP53 is wild type in 25 samples and the rest have TP53 mutations. None contributes to co-existing mutations. Using a conventional transcriptomic analysis, we obtained 394 differentially expressed genes between Group 1 and Group 2 in PAAD samples (Figure 3, 137 upregulated and 257 downregulated genes where p-value<0.01 (Mann Whitney U Test) and |log_2_FC| > 0.5). This allowed us to identify *TP53* mutations (contributing to pairs) in transcriptional regulation. A set of proteins in immune response, positive regulation of cell proliferation and cell-cell signaling are enriched in Group 1 compared to Group 2. We discovered significant upstream transcription factors (TFs) that regulate differentially expressed genes using the TRRUST (version 2) dataset, a manually curated database of human and mouse transcriptional regulatory networks. As a result, 55 TFs are retrieved as the main regulators including *SP1 (*encodes Sp1), *SP3* (encodes Sp3), *NFKD1*, *JUN* and *TP53* (encodes p53) (Figure 3). We constructed the network of the TFs and KRAS with CancerGeneNet [3] and as expected, observed that the proliferation phenotype is upregulated, consistent with *TP53* driver mutations significantly affecting the transcriptional output compared to the single mutant counterparts [4].

**Despite expected oncogene-induced senescence (OIS) cells may survive**

We know that the number of mutations increases during proliferation and over time. Yet, cells survive, suggesting that OIS combinations were sidestepped, or more likely, since somatic mutations emerge sporadically, cells harboring excluded combinations did not endure [5]. Co-expression of *KRAS*^G12D^ and *BRAF*^V600E^, two strong drivers, led to OIS in lung cancer in mouse models [6]. The absence of co-existing *NRAS* and *BRAF* variants, such as *BRAF*^V600E^ in the RTK-RAS pathway in the TCGA skin cutaneous melanoma cohort, illustrates mutual exclusivity. Similarly, *BRAF* and RAS gene family mutations do not co-exist in metastatic colorectal cancer tumors [7]. OIS, a tumor-suppressive mechanism arresting cell cycle progression, can be the main reason for excluding the co-existence of driver mutations in the same — here MAPK— pathway [8-10]. Along similar lines, overexpression of oncogenes such as *KRAS*, *BRAF*, and *MYC*, which also generate strong proliferation signal, may induce OIS, as also may stronger PI3K/AKT signaling. Loss of *PTEN*, which negatively regulates the PI3K/AKT signaling, can trigger OIS through a p53-dependent pathway [11], or via coupling with strong PI3K mutational variants. Both constitutively generate signaling lipid PIP_3_. Examples of mutually exclusive relations between protein pairs include *BRCA2*-*TP53*, *BRCA1*-*PARP1*, and *PTEN*-*PIK3CA* in breast cancer; in ovary cancer *BRCA1*-*CCNE1*, *BRAF*-*KRAS*, *ERBB2*-*KRAS* [12].

Considering the additivity of the mutational impact, strong mutations affecting the same pathway might be mutually exclusive in a tumor to prevent OIS synthetic lethality [13-15], or OIS [6, 16], which is its molecular basis. Some of the *in trans* mutation pairs that are prominent in specific tissues or cancer subtypes are associated with poor survival [17, 18]. Examples include mutated *RNF43* together with *BRAF*^V600E^, which significantly co-exists in our dataset.

**Mutations affecting protein-protein interactions as metastatic markers**

Complex biological functions are mostly managed through protein-protein interactions (PPIs), and mutations impacting them may alter the cell phenotypes [19]. Alterations perturbing PPIs can impact drug outcomes [20-22]. Detection of druggable oncoPPIs [20] is promising since some could constitute more cancer-specific therapeutic targets [23, 24], although the pre-existing mutation load can impact the efficacy [25]. The therapeutic efficacy of most drugs relies on their interactions with proteins, yet current databases predominantly emphasize therapeutic targets, neglecting off-targets. To address this disparity, computational methods are employed to predict drug-protein interactions by leveraging similarities in drug structures and protein profiles [26, 27]. Among the Fujian cohort mutations classified as metastatic markers in gastric carcinomas, one is a *PTPRT* mutation [28], and another, Chondrosarcoma *TERT*  promoter mutation is a metastasis marker [29]. A real-world clinicogenomic dataset permitted the discovery of biomarkers that predict treatment outcomes that affect patients' survival [30]. Treatment-specific genetic alterations in non-small cell lung cancer (NSCLC) include mutually exclusive and co-existing mutations [31, 32]. A study which analyzed molecular and cellular data from 154 patients with lung cancer brain metastases, revealing that brain metastases show greater tumor heterogeneity than primary lung tumors, share key gene mutations (*TTN, TP53*, etc.), exhibit activated mitochondrial metabolism but suppressed immune response, and demonstrated that combining mitochondrial-targeting drugs (gamitrinib) with immunotherapy (anti-PD-1) improved survival in mouse models [33].

**Primary tumors and metastases**

Over 70% of all mutation pairs exist in primary tumors; however, there is a small set that are specific to metastatic samples. Estrogen Receptor 1 (*ESR1*) and *PIK3CA* top the list. They are components of the well-established insulin– phosphoinositide 3-kinase (PI3K) signaling cascade, known to play key roles in cancer cells [34]. *AKT* is in the PI3K/mTOR pathway. *ESR1* and PI3Kα lipid kinase coordinates the glucose metabolism upstream, and *AKT* and mTOR, are protein kinases downstream of PI3K. The pathway feeds into the cell cycle, acting dominantly in protein synthesis and is a driver of malignant transformation. Also ranking high is GATA3, a pioneer transcription factor (TF) [35] controlling multiple proteins in embryonic development known to be markers for some cancers, particularly those related with the breast. *GATA3* acts upstream of FOXA1, also a pioneer TF, in mediating *ESR1* [36]. It is also associated with PI3K/mTOR. Consistently, we observe *GATA3*/*ESR1* and *GATA3*/*PIK3CA* signatures. Additional candidates that we observe are *TP53*, *ERBB2* (a receptor tyrosine kinase with intrinsic tyrosine kinase activity, established to mediate breast cancer) and CDH1 (Cadherin-1 or Epithelial cadherin). These confirm and link to established major mechanisms in proliferative cancers. Wang et al. analyzed genomic alterations in 114 Chinese NSCLC patients to understand metastasis patterns and develop predictive models. The key findings showed that metastatic patients were more likely to be older, with *TP53* being the most frequent mutation, while factors like female gender and certain gene mutations (*EGFR*+, *ASXL2*-, *STK11*-) were associated with better survival outcomes [37]. Yet another study of 1817 *KRAS*-mutant lung adenocarcinoma patients found that mutations in *KEAP1* and *SMARCA4* genes were significantly associated with metastatic disease and poor survival outcomes. Notably, while *STK11* mutations alone weren't linked to metastasis, functional *STK11* loss may contribute to poor outcomes in KEAP1-mutated tumors, with the impact varying by metastatic site [38].

**Limitations of the study**

We recognize several limitations inherent in our study, primarily due to insufficient availability of omics and clinical data to complement the mutation data. Our dataset was restricted to RNA-seq from TCGA samples, constraining our ability to compare gene expression between single-mutant and co-mutant samples across various tissues. The scarcity of congruent cell line and patient-derived xenograft data for single-mutant and co-mutant samples hindered our capacity to explore drug response and tumor growth patterns. Advancing this framework necessitates the application of large, homogeneous, and high-quality datasets, as well as comprehensive RNA-seq profiles alongside clinical data, such as patient survival information. This approach would facilitate a more nuanced understanding uncovering novel tumor vulnerabilities and paving the way for the development of combination therapies.


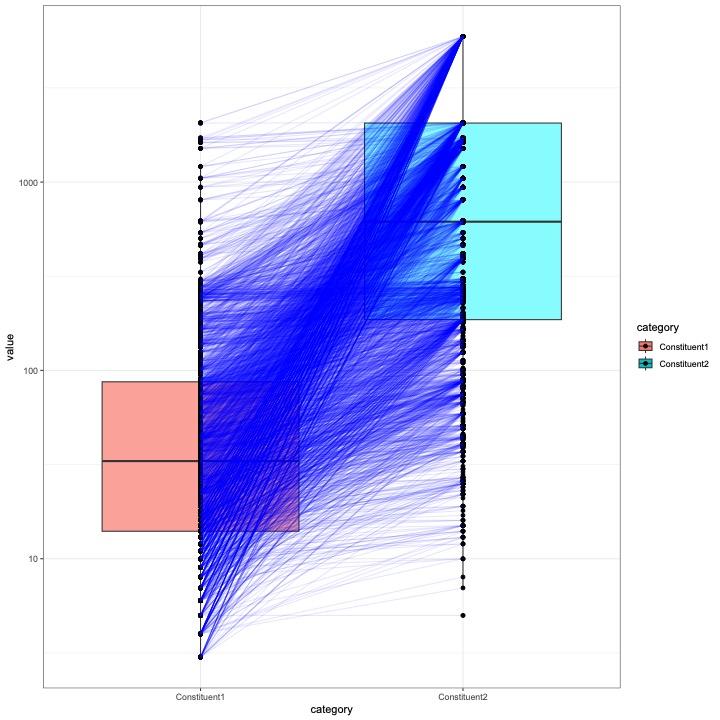


**Fig A. Box plot showing the frequency distribution of double mutation constituents with connected dots.** The lines indicate the co-occurrence of a frequent mutation with a relatively rare one.


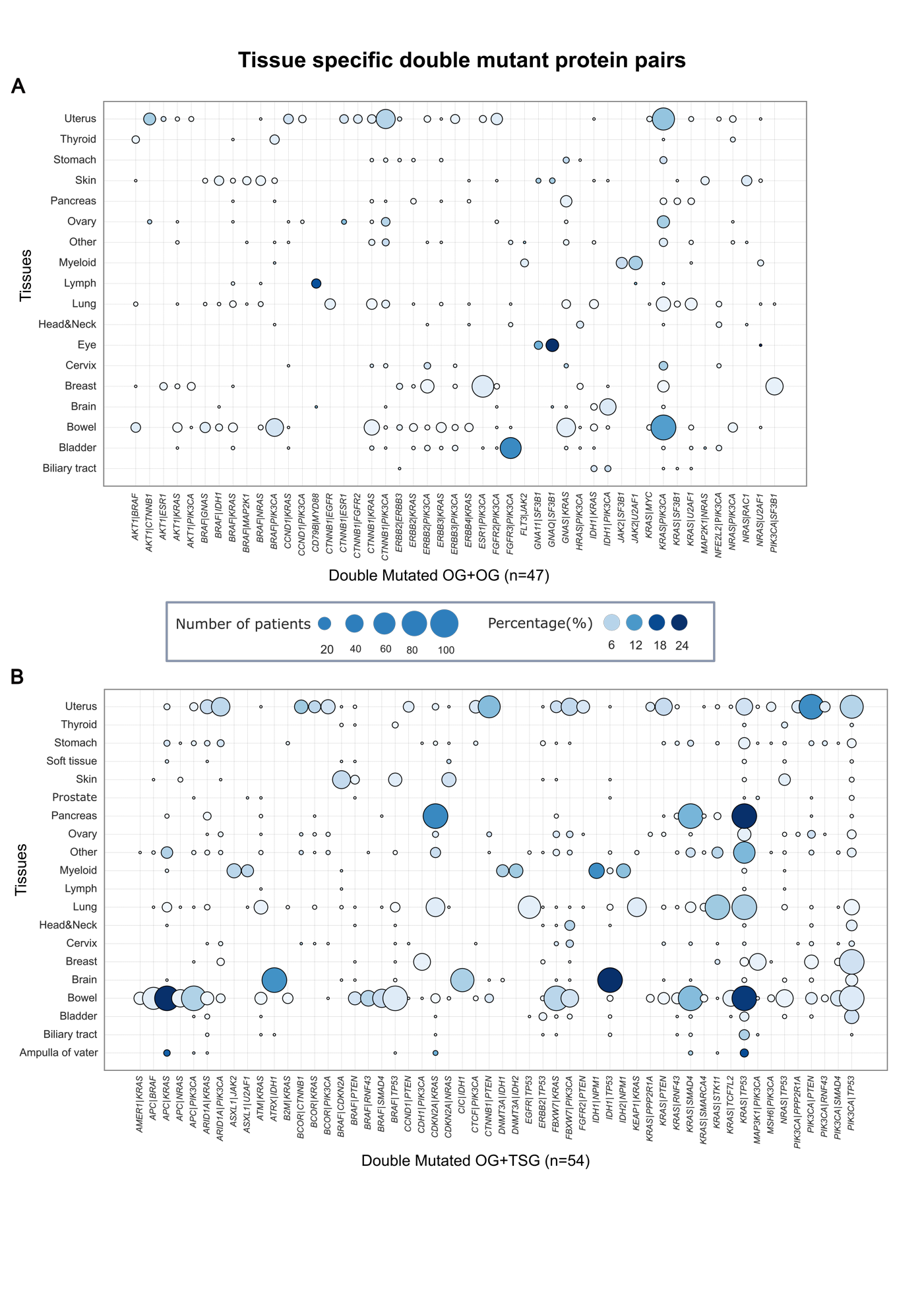


**Fig B. Widespread different gene mutation pairs in different tissues.** We call a protein pair A|B if there is a mutation pair where the component mutations are on proteins A and B, and the protein couple A|B is mutated on the union of tumors with the mutations on these proteins. Node size is the overall number of patients with these mutations in each tissue, and node color is a fraction of all tumors with mutation pairs to all tumors in tissues. **(A)** Different oncogene pairs (mutated on at least 10 patients in a tissue) and their tissue prevalence (have mutation in at least 10 patients on an oncogene couple). Preeminent oncogene pairs show tissue-specific enrichment: *ESR1*|*PIK3CA* in breast (2.5%), *KRAS*|*PIK3CA* in bowel (11%), and both *CTNNB1*|*PIK3CA* (6%) and *KRAS*|*PIK3CA* (8%) in uterine tissue. **(B)** Different gene couples where one constituent is an oncogene, and the other tumor suppressor gene (mutated on at least 20 patients in a tissue) and their tissue prevalence (have mutation in at least 20 patients on a gene couple). For oncogene and tumor suppressor pairs, *PIK3CA*|*TP53* (~4%), *MAP3K1*|*PIK3CA* (1.5) and *CDH1*|*PIK3CA* (~1.5%) are prominent in breast tissue. Similarly, the pairs *KRAS*|*SMAD4* (9%), *KRAS*|*TP53* (33%) and *CDKN2A*|*KRAS (*13%) are enriched in the pancreas tissue. Among oncogene-tumor suppressor pairs, bowel tissue exhibits diverse combinations, while breast tissue shows specific enrichment of *PIK3CA*|*TP53* (4%), *MAP3K1*|*PIK3CA* (1.5%), and *CDH1*|*PIK3CA* (1.5%). Pancreatic tissue is particularly enriched for KRAS-containing pairs: *KRAS*|*SMAD4* (9%), *KRAS*|*TP53* (33%), and *CDKN2A*|*KRAS* (13%).


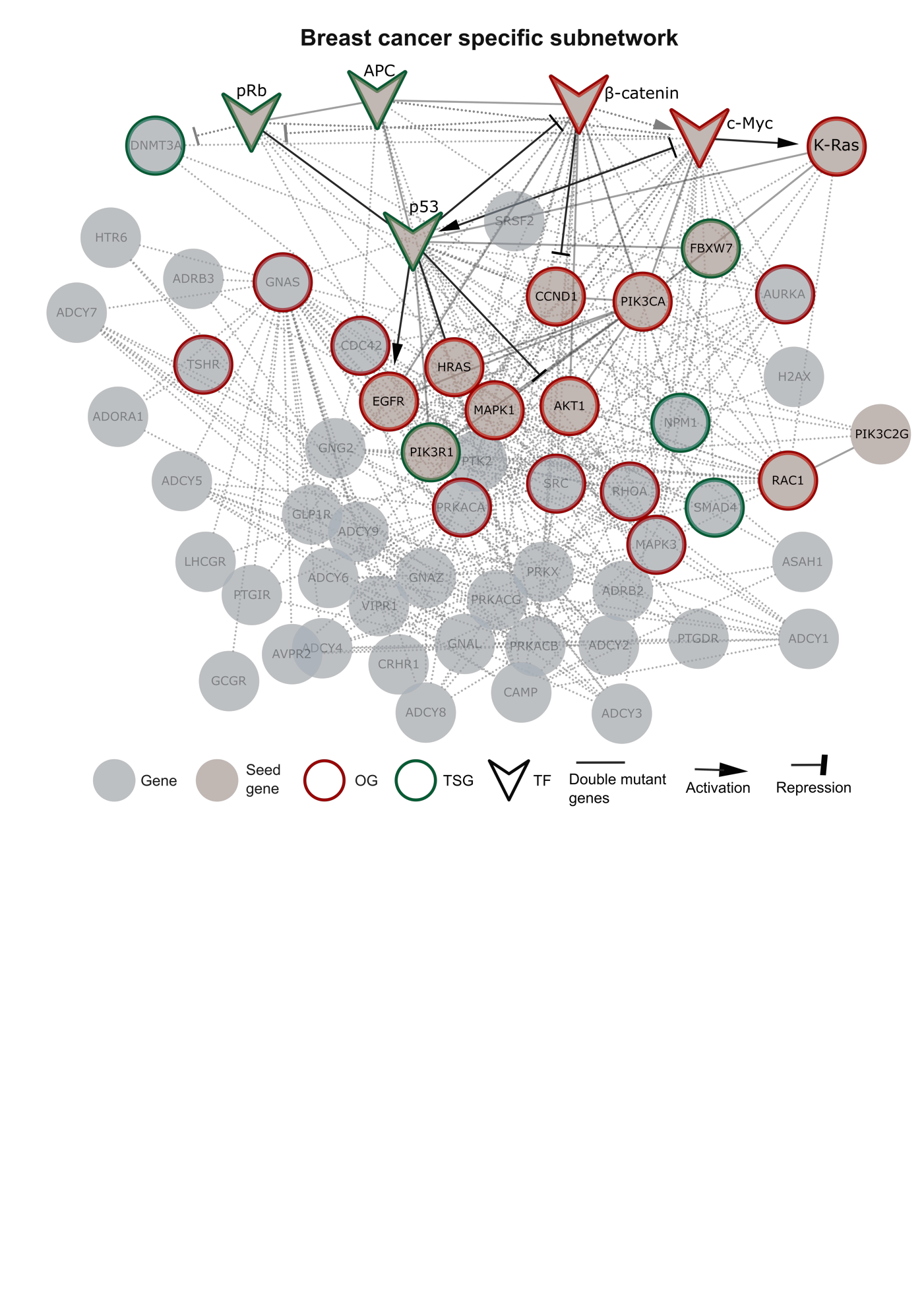


**Fig C. Breast cancer specific subnetwork.** Breast cancer specific subnetwork obtained with the Page Rank algorithm from Omni Path with the seed proteins that have mutation pairs and the tissue specific fraction is greater than 0.5. Blue nodes are the proteins from the PPI, pink nodes are the seed proteins which are mutation pair components. Transcription factors (TFs) are V-shaped nodes. Border color is green if the gene is tumor suppressor gene and red if oncogene. The edges are solid lines if there is an edge between nodes in the PPI that contribute to a mutation pair. Dashed lines depict the direct interaction in the PPI.


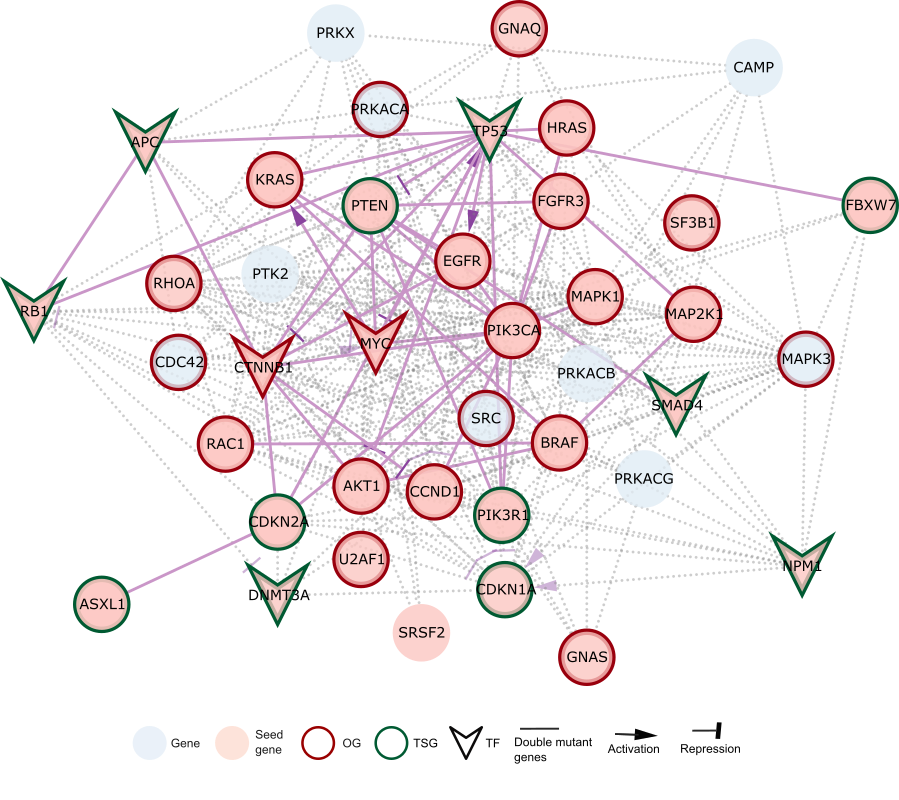


**Fig D. Pancreas cancer specific subnetwork.** There are 40 nodes 267 edges in the subnetwork obtained from Omni Path PPI by Page Rank Algorithm with 23 seed genes.


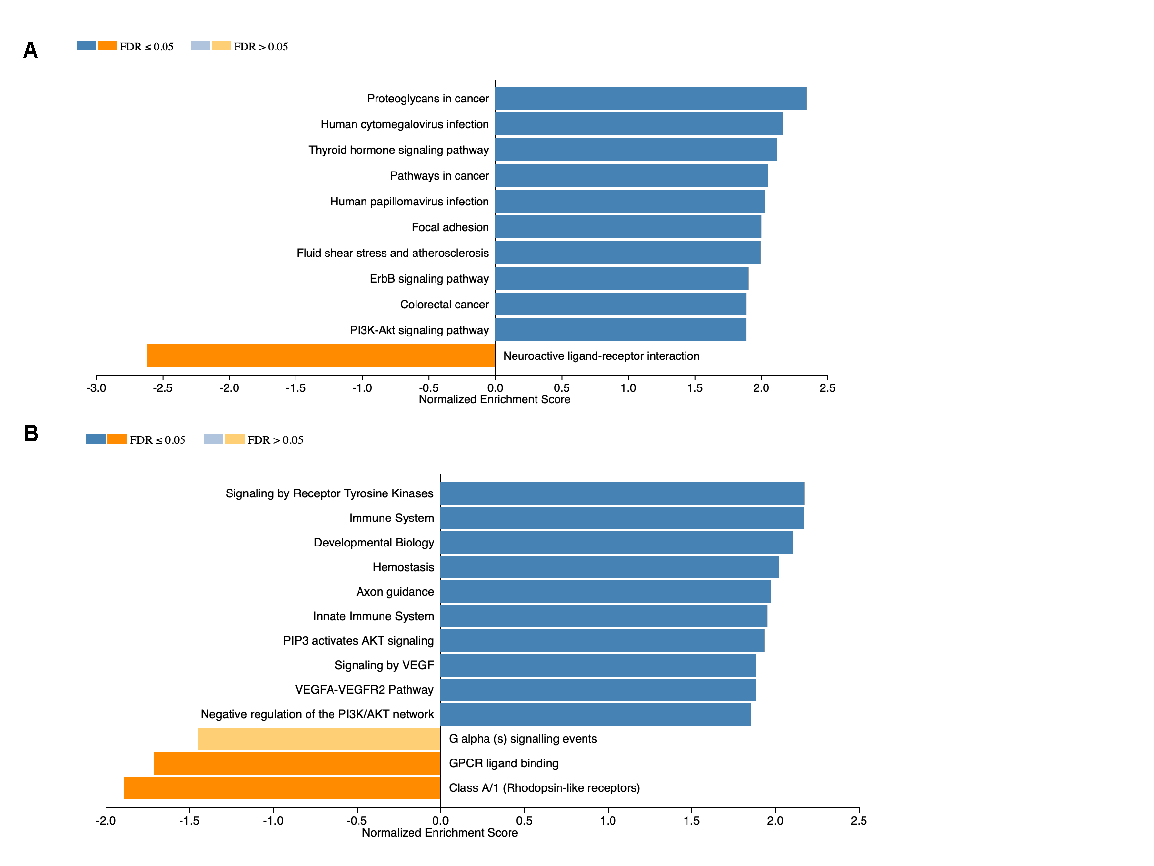


**Fig E.** **Enriched pathways in breast cancer.** Gene set enrichment analysis of breast cancer specific subnetwork in (A) KEGG , (B) Reactome.


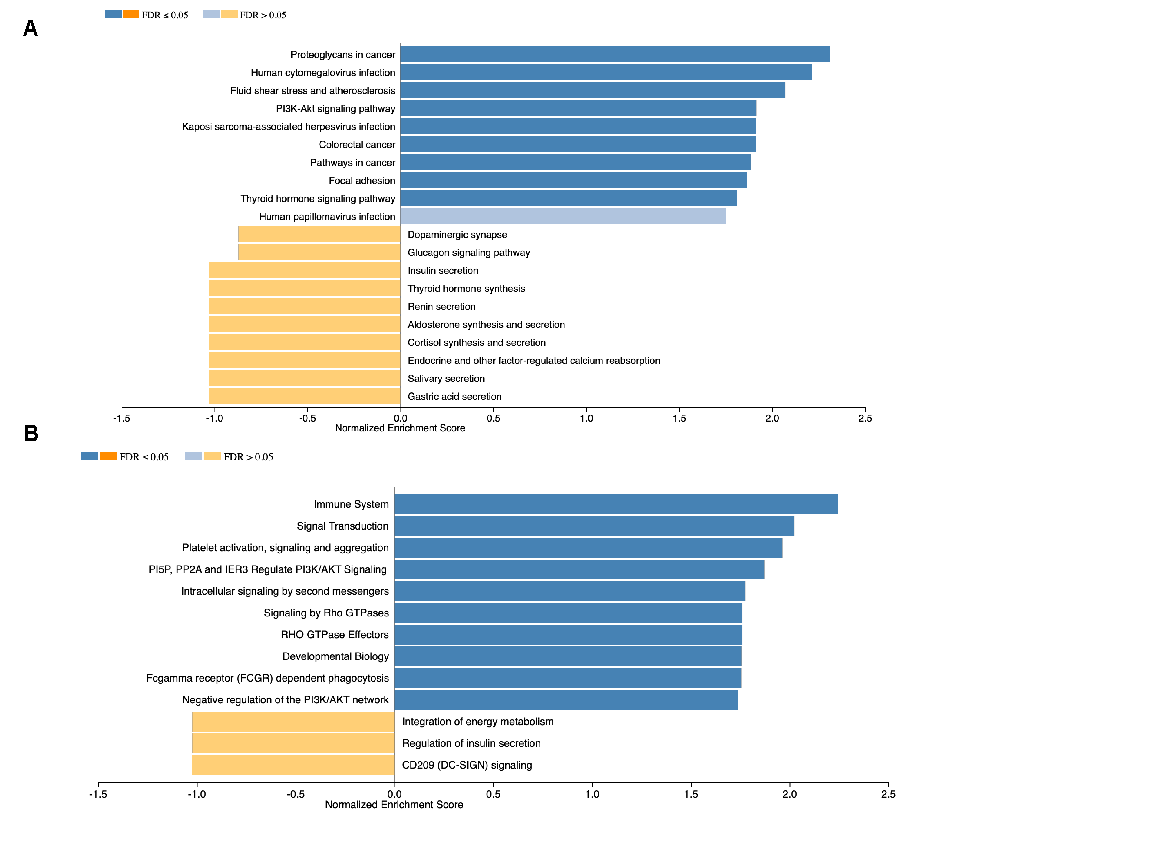


**Fig F. Enriched pathways in pancreas cancer.** Gene set enrichment analysis of pancreas cancer specific subnetwork in (A) KEGG, (B) Reactome.


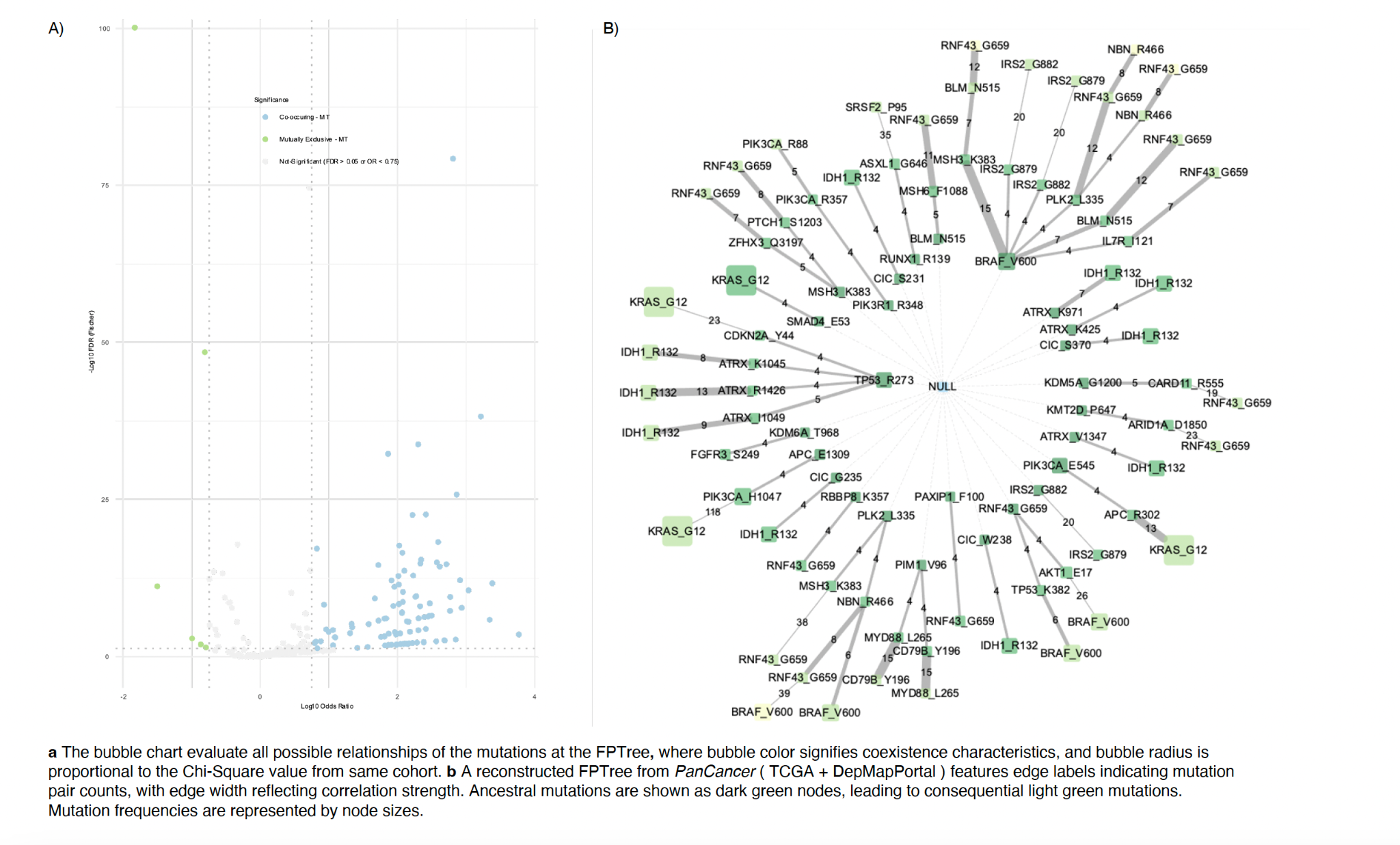


**Fig G. Pan cancer metastatic tree (A**)The Volcano plot of pairs of same cohort, log10 Odds Ratio against -log10 Fisher’s p-value, with non-significant pairs (σ > 0.05, |Odds Ratio| > 0.75) in gray. **(B)** reconstructed FPTree from pan-cancer metastatic cohort features edge labels indicating mutation pair counts, with edge width reflecting correlation strength. The tree was constructed using a minimum support threshold of 7×10⁻⁵ and a minimum confidence threshold of 0.95. Ancestral mutations are shown as dark green nodes, leading to consequential light green mutations. Mutation frequencies are represented by node sizes.


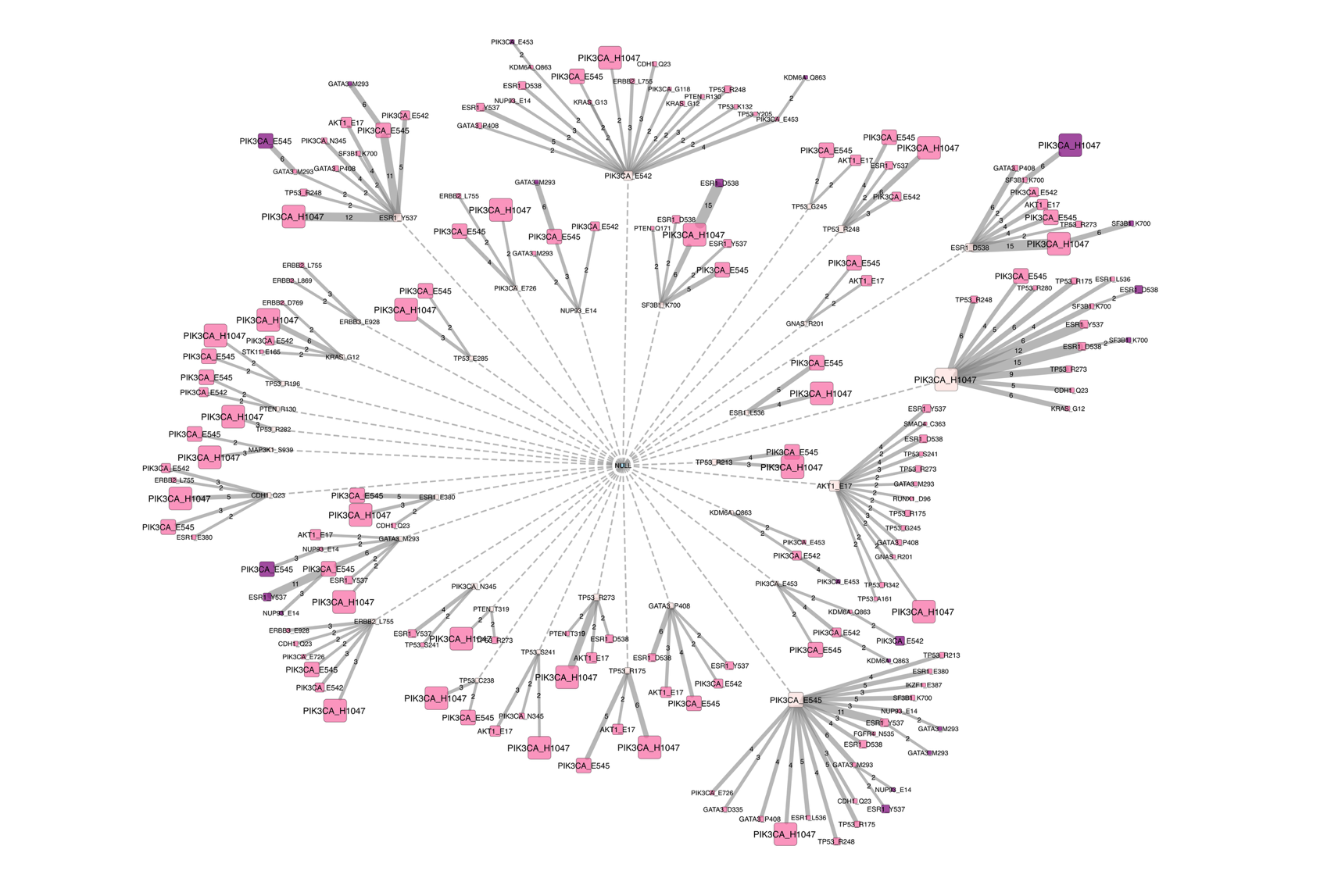


**Fig H. Breast cancer mutation co-occurrence tree including triplets.** The tree illustrates doublet, and triplet co-mutation patterns among recurrent driver mutations in metastatic breast cancer. Due to the limited cohort size, we used relaxed construction parameters (MinSupport = 0.0007, MinTresh = 0.01), resulting in a denser tree with more nodes and edges. To preserve structural clarity pairs directly attached to the null root were excluded, as they offer limited hierarchical information.

**References**

1. Kanehisa M, Goto S. KEGG: kyoto encyclopedia of genes and genomes. Nucleic Acids Res. 2000;28(1):27-30. doi: 10.1093/nar/28.1.27. PubMed PMID: 10592173; PubMed Central PMCID: PMCPMC102409.

2. Gu Z, Hubschmann D. Make Interactive Complex Heatmaps in R. Bioinformatics. 2022;38(5):1460-2. doi: 10.1093/bioinformatics/btab806. PubMed PMID: 34864868; PubMed Central PMCID: PMCPMC8826183.

3. Iannuccelli M, Micarelli E, Surdo PL, Palma A, Perfetto L, Rozzo I, et al. CancerGeneNet: linking driver genes to cancer hallmarks. Nucleic Acids Res. 2020;48(D1):D416-D21. doi: 10.1093/nar/gkz871. PubMed Central PMCID: PMCPMC6943052.

4. Gorelick AN, Sánchez-Rivera FJ, Cai Y, Bielski CM, Biederstedt E, Jonsson P, et al. Phase and context shape the function of composite oncogenic mutations. Nature. 2020;582(7810):100-3. doi: 10.1038/s41586-020-2315-8. PubMed Central PMCID: PMCPMC7294994.

5. Nussinov R, Jang H, Tsai C-J, Cheng F. Precision medicine review: rare driver mutations and their biophysical classification. Biophys Rev. 2019;11(1):5-19. doi: 10.1007/s12551-018-0496-2. PubMed Central PMCID: PMCPMC6381362.

6. Cisowski J, Bergo MO. What makes oncogenes mutually exclusive? Small GTPases. 2017;8(3):187-92. doi: 10.1080/21541248.2016.1212689. PubMed Central PMCID: PMCPMC5584735.

7. Ahronian LG, Sennott EM, Van Allen EM, Wagle N, Kwak EL, Faris JE, et al. Clinical Acquired Resistance to RAF Inhibitor Combinations in BRAF-Mutant Colorectal Cancer through MAPK Pathway Alterations. Cancer Discov. 2015;5(4):358-67. doi: 10.1158/2159-8290.CD-14-1518. PubMed Central PMCID: PMCPMC4390490.

8. Wang B, Kohli J, Demaria M. Senescent Cells in Cancer Therapy: Friends or Foes? Trends Cancer Res. 2020;6(10):838-57. doi: 10.1016/j.trecan.2020.05.004.

9. Liu X-L, Ding J, Meng L-H. Oncogene-induced senescence: a double edged sword in cancer. Acta Pharmacol Sin. 2018;39(10):1553-8. doi: 10.1038/aps.2017.198. PubMed Central PMCID: PMCPMC6289471.

10. Schmitt CA, Wang B, Demaria M. Senescence and cancer - role and therapeutic opportunities. Nat Rev Clin Oncol. 2022;19(10):619-36. Epub 20220831. doi: 10.1038/s41571-022-00668-4. PubMed PMID: 36045302; PubMed Central PMCID: PMCPMC9428886.

11. Jung SH, Hwang HJ, Kang D, Park HA, Lee HC, Jeong D, et al. mTOR kinase leads to PTEN-loss-induced cellular senescence by phosphorylating p53. Oncogene. 2019;38(10):1639-50. doi: 10.1038/s41388-018-0521-8. PubMed Central PMCID: PMCPMC6755978.

12. Deng Y, Luo S, Deng C, Luo T, Yin W, Zhang H, et al. Identifying mutual exclusivity across cancer genomes: computational approaches to discover genetic interaction and reveal tumor vulnerability. Brief Bioinform. 2019;20(1):254-66. doi: 10.1093/bib/bbx109.

13. van de Haar J, Canisius S, Yu MK, Voest EE, Wessels LFA, Ideker T. Identifying Epistasis in Cancer Genomes: A Delicate Affair. Cell. 2019;177(6):1375-83. doi: 10.1016/j.cell.2019.05.005. PubMed Central PMCID: PMCPMC6816465.

14. Sanchez-Vega F, Mina M, Armenia J, Chatila WK, Luna A, La KC, et al. Oncogenic Signaling Pathways in The Cancer Genome Atlas. Cell. 2018;173(2):321-37.e10. doi: 10.1016/j.cell.2018.03.035. PubMed Central PMCID: PMCPMC6070353.

15. Waarts MR, Stonestrom AJ, Park YC, Levine RL. Targeting mutations in cancer. J Clin Invest. 2022;132(8). doi: 10.1172/JCI154943. PubMed PMID: 35426374; PubMed Central PMCID: PMCPMC9012285.

16. Nussinov R, Tsai C-J, Jang H. A New View of Activating Mutations in Cancer. Cancer Res. 2022;82(22):4114-23. doi: 10.1158/0008-5472.CAN-22-2125. PubMed Central PMCID: PMCPMC9664134.

17. Matsumoto A, Shimada Y, Nakano M, Oyanagi H, Tajima Y, Nakano M, et al. RNF43 mutation is associated with aggressive tumor biology along with BRAF V600E mutation in right-sided colorectal cancer. Oncol Rep. 2020;43(6):1853-62. doi: 10.3892/or.2020.7561. PubMed Central PMCID: PMCPMC7160543.

18. De P, Dey N. Mutation-Driven Signals of ARID1A and PI3K Pathways in Ovarian Carcinomas: Alteration Is An Opportunity. Int J Mol Sci. 2019;20(22). doi: 10.3390/ijms20225732. PubMed Central PMCID: PMCPMC6888220.

19. Jubb HC, Pandurangan AP, Turner MA, Ochoa-Montaño B, Blundell TL, Ascher DB. Mutations at protein-protein interfaces: Small changes over big surfaces have large impacts on human health. Prog Biophys Mol Biol. 2017;128:3-13. doi: 10.1016/j.pbiomolbio.2016.10.002.

20. Cheng F, Zhao J, Wang Y, Lu W, Liu Z, Zhou Y, et al. Comprehensive characterization of protein-protein interactions perturbed by disease mutations. Nat Genet. 2021;53(3):342-53. doi: 10.1038/s41588-020-00774-y. PubMed Central PMCID: PMCPMC8237108.

21. Cheng F, Liang H, Butte AJ, Eng C, Nussinov R. Personal Mutanomes Meet Modern Oncology Drug Discovery and Precision Health. Pharmacol Rev. 2019;71(1):1-19. doi: 10.1124/pr.118.016253. PubMed Central PMCID: PMCPMC6294046.

22. Nussinov R, Jang H, Tsai C-J, Cheng F. Review: Precision medicine and driver mutations: Computational methods, functional assays and conformational principles for interpreting cancer drivers. PLoS Comput Biol. 2019;15(3):e1006658. doi: 10.1371/journal.pcbi.1006658. PubMed Central PMCID: PMCPMC6438456.

23. Ivanov AA. Explore Protein-Protein Interactions for Cancer Target Discovery Using the OncoPPi Portal. Methods Mol Biol. 2020;2074:145-64. doi: 10.1007/978-1-4939-9873-9_12.

24. Ivanov AA, Revennaugh B, Rusnak L, Gonzalez-Pecchi V, Mo X, Johns MA, et al. The OncoPPi Portal: an integrative resource to explore and prioritize protein-protein interactions for cancer target discovery. Bioinformatics. 2018;34(7):1183-91. doi: 10.1093/bioinformatics/btx743. PubMed Central PMCID: PMCPMC6030952.

25. Nussinov R, Tsai C-J, Jang H. Anticancer drug resistance: An update and perspective. Drug Resist Updat. 2021;59:100796. doi: 10.1016/j.drup.2021.100796. PubMed Central PMCID: PMCPMC8810687.

26. Wang C, Kurgan L. Review and comparative assessment of similarity-based methods for prediction of drug-protein interactions in the druggable human proteome. Brief Bioinform. 2019;20(6):2066-87. doi: 10.1093/bib/bby069. PubMed PMID: 30102367.

27. Wang C, Kurgan L. Survey of Similarity-Based Prediction of Drug-Protein Interactions. Curr Med Chem. 2020;27(35):5856-86. doi: 10.2174/0929867326666190808154841. PubMed PMID: 31393241.

28. Chen C, Shi C, Huang X, Zheng J, Zhu Z, Li Q, et al. Molecular Profiles and Metastasis Markers in Chinese Patients with Gastric Carcinoma. Sci Rep. 2019;9(1):13995. doi: 10.1038/s41598-019-50171-7. PubMed Central PMCID: PMCPMC6769015.

29. Zhang Y, Chen Y, Yang C, Seger N, Hesla AC, Tsagkozis P, et al. TERT promoter mutation is an objective clinical marker for disease progression in chondrosarcoma. Mod Pathol. 2021;34(11):2020-7. doi: 10.1038/s41379-021-00848-0. PubMed Central PMCID: PMCPMC8514332.

30. Liu R, Rizzo S, Waliany S, Garmhausen MR, Pal N, Huang Z, et al. Systematic pan-cancer analysis of mutation-treatment interactions using large real-world clinicogenomics data. Nat Med. 2022;28(8):1656-61. doi: 10.1038/s41591-022-01873-5.

31. Smith MR, Wang Y, D'Agostino R, Jr., Liu Y, Ruiz J, Lycan T, et al. Prognostic Mutational Signatures of NSCLC Patients treated with chemotherapy, immunotherapy and chemoimmunotherapy. NPJ Precis Oncol. 2023;7(1):34. doi: 10.1038/s41698-023-00373-0. PubMed Central PMCID: PMCPMC10042886.

32. Zhang F, Wang J, Xu Y, Cai S, Li T, Wang G, et al. Co-occurring genomic alterations and immunotherapy efficacy in NSCLC. NPJ Precis Oncol. 2022;6(1):4. doi: 10.1038/s41698-021-00243-7. PubMed Central PMCID: PMCPMC8766442.

33. Duan H, Ren J, Wei S, Yang Z, Li C, Wang Z, et al. Integrated analyses of multi-omic data derived from paired primary lung cancer and brain metastasis reveal the metabolic vulnerability as a novel therapeutic target. Genome Med. 2024;16(1):138. Epub 20241126. doi: 10.1186/s13073-024-01410-8. PubMed PMID: 39593114; PubMed Central PMCID: PMCPMC11590298.

34. Hopkins BD, Goncalves MD, Cantley LC. Insulin-PI3K signalling: an evolutionarily insulated metabolic driver of cancer. Nat Rev Endocrinol. 2020;16(5):276-83. doi: 10.1038/s41574-020-0329-9. PubMed Central PMCID: PMCPMC7286536.

35. Tanaka H, Takizawa Y, Takaku M, Kato D, Kumagawa Y, Grimm SA, et al. Interaction of the pioneer transcription factor GATA3 with nucleosomes. Nat Commun. 2020;11(1):4136. doi: 10.1038/s41467-020-17959-y. PubMed Central PMCID: PMCPMC7434886.

36. Theodorou V, Stark R, Menon S, Carroll JS. GATA3 acts upstream of FOXA1 in mediating ESR1 binding by shaping enhancer accessibility. Genome Res. 2013;23(1):12-22. doi: 10.1101/gr.139469.112. PubMed Central PMCID: PMCPMC3530671.

37. Wang K, Ye M, Mo Z, Huang X, Li Y, Wei S. Comprehensive analysis of genomic alterations and novel prognostic biomarkers, and establishment of prediction models of metastasis in metastatic non-small cell lung cancer. J Cancer. 2025;16(1):339-50. Epub 20250101. doi: 10.7150/jca.97070. PubMed PMID: 39744567; PubMed Central PMCID: PMCPMC11660132.

38. Boiarsky D, Lydon CA, Chambers ES, Sholl LM, Nishino M, Skoulidis F, et al. Molecular markers of metastatic disease in KRAS-mutant lung adenocarcinoma. Ann Oncol. 2023;34(7):589-604. Epub 20230429. doi: 10.1016/j.annonc.2023.04.514. PubMed PMID: 37121400; PubMed Central PMCID: PMCPMC10425882.
